# Genome-wide analysis of health-related biomarkers in the UK Household Longitudinal Study reveals novel associations

**DOI:** 10.1038/s41598-017-10812-1

**Published:** 2017-09-08

**Authors:** Bram P. Prins, Karoline B. Kuchenbaecker, Yanchun Bao, Melissa Smart, Delilah Zabaneh, Ghazaleh Fatemifar, Jian’an Luan, Nick J. Wareham, Robert A. Scott, John R. B. Perry, Claudia Langenberg, Michaela Benzeval, Meena Kumari, Eleftheria Zeggini

**Affiliations:** 1 Wellcome Trust Sanger Institute, Hinxton, UK; 2 Institute for Social and Economic Research, University of Essex, Wivenhoe Park, Colchester, Essex UK; 3 MRC Social, Genetic & Developmental Psychiatry Centre, IoPPN, KCL, London, UK; 4 Institute for Health Informatics, UCL and the Farr Institute of Health Informatics, London, UK; 5 MRC Epidemiology Unit, University of Cambridge School of Clinical Medicine, Box 285 Institute of Metabolic Science, Cambridge Biomedical Campus, Cambridge, UK

## Abstract

Serum biomarker levels are associated with the risk of complex diseases. Here, we aimed to gain insights into the genetic architecture of biomarker traits which can reflect health status. We performed genome-wide association analyses for twenty serum biomarkers involved in organ function and reproductive health. 9,961 individuals from the UK Household Longitudinal Study were genotyped using the Illumina HumanCoreExome array and variants imputed to the 1000 Genomes Project and UK10K haplotypes. We establish a polygenic heritability for all biomarkers, confirm associations of fifty-four established loci, and identify five novel, replicating associations at genome-wide significance. A low-frequency variant, rs28929474, (beta = 0.04, P = 2 × 10^−10^) was associated with levels of alanine transaminase, an indicator of liver damage. The variant is located in the gene encoding serine protease inhibitor, low levels of which are associated with alpha-1 antitrypsin deficiency which leads to liver disease. We identified novel associations (rs78900934, beta = 0.05, P = 6 × 10^−12^; rs2911280, beta = 0.09, P = 6 × 10^−10^) for dihydroepiandrosterone sulphate, a precursor to major sex-hormones, and for glycated haemoglobin (rs12819124, beta = −0.03, P = 4 × 10^−9^; rs761772, beta = 0.05, P = 5 × 10^−9^). rs12819124 is nominally associated with risk of type 2 diabetes. Our study offers insights into the genetic architecture of well-known and less well-studied biomarkers.

## Introduction

Serum biomarker levels are associated with the risk of complex diseases and are therefore increasingly used in clinical practice to assist with diagnosis, status monitoring and disease management. Well-known examples include the measurement of lipid levels in the context of cardiovascular disease or liver enzymes and albumin to assess liver function.

Serum biomarker levels have a polygenic basis. As demonstrated in the case of lipids, identifying genetic associations can provide new insights into disease aetiology which can in turn guide drug discovery and be useful for diagnosis and risk stratification^1–3^. However, the genetic architecture of most health-related biomarkers has not been studied as extensively as for lipids. Alleles identified to be associated with protein biomarkers to date are predominantly common (minor allele frequency (MAF) >5%). This is primarily driven by genotyping technology and composition of arrays or imputation reference panels used to date^4–6^. Systematically evaluating the association of low frequency and rare variants can provide new insights regarding the genetic architecture of protein biomarkers.

The importance of studying the joint impact of genetic and non-genetic factors on health has been recognised by the UK Household Longitudinal Study (UKHLS, www.understandingsociety.ac.uk), also known as Understanding Society. Involving a total of 40,000 households representative of the UK population, UKHLS is the largest panel survey in the world to support social research. A wide range of social, economic, environment, behavioural, attitudinal, physiological and biomedical variables, including a large panel of the most commonly used clinical biomarkers, have been measured for a representative selection of the sample. This study represents a large sample with very homogenous biomarker measurements, in which recruitment and processing have been carried out consistently and following strict protocols.

Here we describe genome-wide investigation of associations with 20 biomarkers relevant to blood clot formation (fibrinogen), diabetic status (glycated haemoglobin [HbA1c]), insulin-like growth factor 1 [IGF-1]), inflammation (C-reactive protein [CRP]), iron homeostasis (ferritin, haemoglobin), lipid metabolism (HDL-, LDL- and total cholesterol, triglycerides), liver function (alanine and aspartate transaminase, alkaline phosphatase, gamma glutamyl transferase [GGT]), liver and kidney function (albumin, creatinine, eGFR, urea), and reproductive health (dihydroepiandrosterone sulphate [DHEAS], testosterone) in 9,961 individuals from UKHLS. We also leverage the homogeneity of the sample and its size to estimate the narrow sense heritability which has not yet been quantified for many of these biomarkers.

## Results

### Imputation and genomic coverage

After quality control, genotype data for 525,314 variants were available for 9,961 individuals (Table 1). Following imputation based on the combined reference panel of UK10K and 1000 Genomes Project phase 3, we analysed 23,756,480 variants with imputation accuracy >0.4. Of those, 14,364,872 were rare (MAF <1%, minor allele count (MAC) >10) (2,237,400 of which had imputation accuracy >0.9), 2,732,394 low-frequency (1%≤ MAF <5%) and 6,659,214 common (MAF ≥5%).

**Table 1:** Descriptive statistics for the sample and the measured biomarkers.

Variable	units	N missing	Female					Male				
			N	mean	IQR*	min	max	N	mean	IQR*	min	max
Age	years	0	5574	52.1	25	16	99	4387	52.82	25	16	97
BMI		285	5416	28.02	7.4	14.5	75.7	4260	28.09	5.6	15.8	66.5
Albumin	G/L	137	5501	46.24	4	36	57	4323	47.48	4	36	57
Alkaline Phosphatase	lu/L	228	5451	70.99	26	22	191	4282	71.86	24	22	217
Alanine Transaminase	lu/L	230	5458	23.66	10	5	152	4273	32.23	16	5	150
Aspartate Transaminase	lu/L	498	5321	28.09	8	13	84	4142	32.12	9	12	82
Fibrinogen	G/L	199	5468	2.87	0.7	1.5	5.2	4294	2.76	0.7	1.5	5.2
Total Cholesterol	Mmol/L	144	5495	5.49	1.5	2.2	10	4322	5.29	1.6	2	10
Dihydroepiandrosterone Sulphate	Umol/L	239	5414	3.76	3.3	0.4	19	4308	5.67	4.9	0.4	25.3
Creatinine	Umol/L	158	5497	68.27	14	33	173	4306	85.9	17	44	178
Gamma Glutamyl Transferase	lu/L	214	5467	27.33	16	5	382	4280	39.75	25	5	368
Glycated haemoglobin	Mmol/mol	525	5288	36.05	6	15	57	4148	36.56	6	18	57
HDL cholesterol	Mmol/L	165	5482	1.68	0.5	0.5	3.4	4314	1.37	0.5	0.4	3.4
Haemoglobin	G/L	294	5392	130.49	13	82	174	4275	145.62	14	84	185
C-Reactive Protein (hs assay)	Mg/L	420	5350	3.53	3	0.2	115.5	4191	3	2.2	0.2	104.9
Insulin-like growth factor 1	Nmol/L	229	5455	17.74	8	2	47	4277	18.43	8	3	47
Ferritin	G/L	143	5499	92.93	82	3	1292	4319	189.11	143	7	3044
Testosterone (for males only)	Nmol/L	5702	NA	NA	NA	NA	NA	4259	15.59	7.3	2.9	40.1
Triglycerides	Mmol/L	216	5482	1.58	1	0.3	6.3	4263	1.99	1.3	0.3	6.3
Urea	Mmol/L	143	5498	5.94	2	2.2	16.5	4320	6.53	2	2.1	16.5

^*^IQR = inter quartile range.

### Heritability and genetic overlap analyses

**Figure 1:**
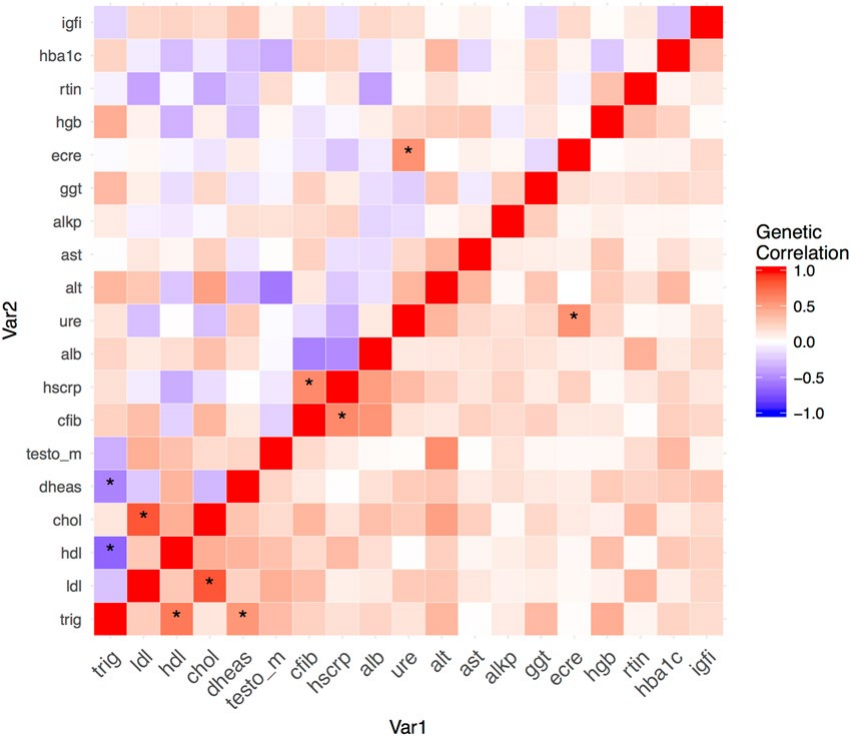
Genetic correlations between different biomarker levels. Colour-coding indicates the strength of the correlations. The lower triangle uses only the red color-coding to make it easier to compare the strength of all correlations. Stars indicate statistically significant associations. Albumin: alb, Alkaline Phosphatase: alkp, Alanine Transaminase: alt, Aspartate Transaminase: ast, Fibrinogen: cfib, Total Cholesterol: chol, LDL cholesterol: ldl, Dihydroepiandrosterone Sulphate: dheas, Creatinine: ecre, Gamma Glutamyl Transferase: ggt, Glycated haemoglobin: hba1c, HDL cholesterol: hdl, Haemoglobin: hgb, C-Reactive Protein: hscrp, Insulin-like growth factor 1: igfi, Ferritin: rtin, Testosterone: testo, Triglycerides: trig, Urea: ure.

For all biomarkers except overall and LDL-cholesterol, alanine transaminase and ferritin there was significant (p < 3.6 × 10^−3^) evidence for a heritable polygenic component (Table 2). Alkaline phosphatase and testosterone had the highest array heritability estimates with h^2^ = 27.7% (standard error (SE): 0.040) and h^2^ = 27.1% (SE: 0.084), respectively. Creatinine, GGT, HbA1c, HDL, IGF1, and triglycerides all had estimates larger than 0.20 while the lowest estimate was observed for ferritin (h^2^ = 6.1%, SE: 0.037). We found statistically significant (p < 5.5 × 10^−4^) evidence of genome-wide pleiotropy between different biomarkers (Fig. 1). There was genetic correlation between lipid biomarkers: triglyceride and HDL-cholesterol levels (genetic correlation rg = −0.67, p = 9.9 × 10^−18^). Triglyceride levels were also inversely genetically linked with DHEAS (rg = −0.53 p = 4.0 × 10^−4^). The genetic correlation between two markers of inflammation, C-reactive protein and fibrinogen, was also significant (rg = 0.60 p = 3.2 × 10^−8^). Finally, the genetic factors for creatinine and urea were positively correlated (rg = 0.56 p = 1.2 × 10^−5^).

**Table 2:** Array heritability (h^2^) estimates and standard errors for 20 biomarkers.

biomarker name	h^2^	standard error	p-value
Albumin	0.15	0.04	8.9 × 10^−6^
Alkaline Phosphatase	0.28	0.04	2.2 × 10^−13^
Alanine Transaminase	0.09	0.04	6.8 × 10^−3^
Aspartate Transaminase	0.09	0.04	2.9 × 10^−3^
Fibrinogen	0.17	0.04	6.5 × 10^−6^
Total Cholesterol	0.07	0.04	0.023
Dihydroepiandrosterone Sulphate	0.17	0.04	4.7 × 10^−6^
in men	0.14	0.08	0.045
in women	0.20	0.07	1.6 × 10^−3^
Creatinine	0.21	0.04	5.2 × 10^−9^
eGFR	0.12	0.04	9.0 × 10^−4^
Gamma Glutamyl Transferase	0.22	0.04	2.4 × 10^−9^
Glycated haemoglobin	0.22	0.04	2.8 × 10^−9^
HDL cholesterol	0.23	0.04	5.9 × 10^−10^
LDL cholesterol	0.08	0.04	0.013
Haemoglobin	0.17	0.04	5.5 × 10^−7^
C-Reactive Protein (hs assay)	0.16	0.04	1.1 × 10^−5^
Insulin-like growth factor 1	0.20	0.04	4.6 × 10^−9^
Ferritin	0.06	0.04	0.043
Testosterone (for males only)	0.27	0.08	4.8 × 10^−4^
Triglycerides	0.23	0.04	3.6 × 10^−10^
Urea	0.14	0.04	2.1 × 10^−4^

### Genome-wide association analyses

**Figure 2:**
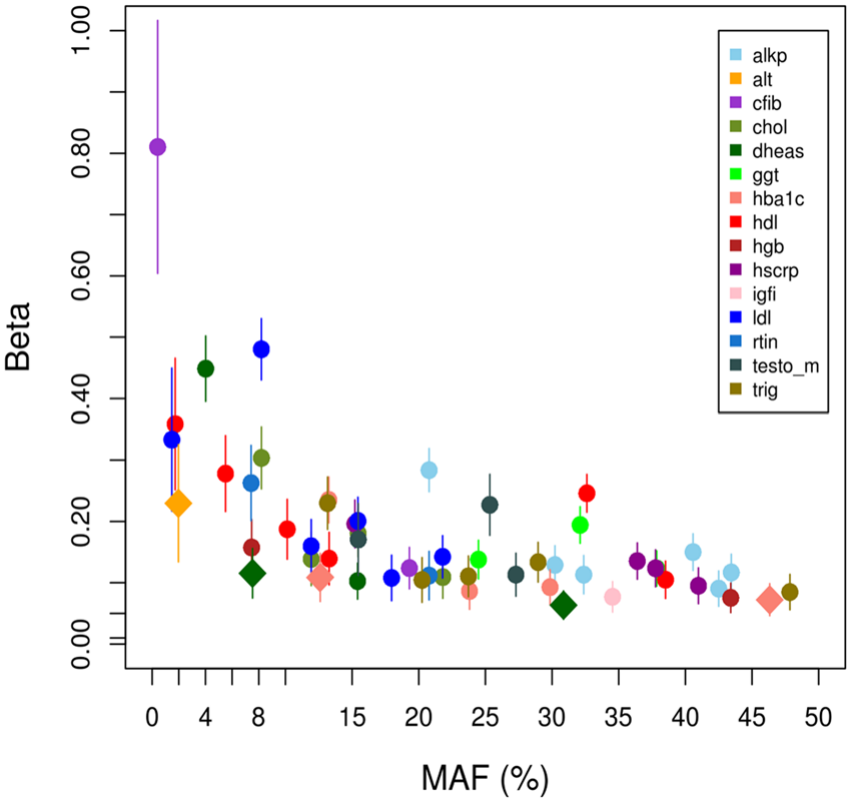
Scatter plot of effect size by frequency of genome-wide significant variants. Effect sizes and 95% confidence intervals (absolute value of beta, expressed in standard deviation units) as a function of minor allele frequencies (MAF), based on the discovery stage of this study. Novel variants (Table 1) are displayed as diamonds, whilst known variants that reach genome-wide significance (P<3.56 × 10^-9^, two-sided) in the discovery stages are display as circles. Alkaline Phosphatase: alkp, Alanine Transaminase: alt, Fibrinogen: cfib, Total Cholesterol: chol, LDL cholesterol: ldl, Dihydroepiandrosterone Sulphate: dheas, Gamma Glutamyl Transferase: ggt, Glycated haemoglobin: hba1c, HDL cholesterol: hdl, Haemoglobin: hgb, C-Reactive Protein: hscrp, Insulin-like growth factor 1: igfi, Ferritin: rtin, Testosterone: testo_m, Triglycerides: trig.

The genome-wide significance threshold of P < 3.56 × 10^−9^ for this study was derived by taking the conventional genome-wide significance threshold (P < 5 × 10^−8^) divided by the effective number of independent traits analysed (N = 14.05, details in Methods). Across fifteen biomarkers, we observed associations of 54 previously reported loci at this threshold (Fig. 2). This includes a low frequency variant, rs148685782 at 4q31 in the fibrinogen gamma chain gene (weighted effect allele frequency [WEAF] = 0.4%, beta[SE] = −0.18[0.02], P = 4.0 × 10^−21^), associated with levels of fibrinogen, a glycoprotein that assists in the blood clot formation. This variant is a missense mutation and has been previously reported to be associated with fibrinogen levels^7^ as well as with hypofibrinogenemia and haemorrhage^8–10^.

From the discovery phase we carried forward 573 independent (pairwise r^2^ < 0.01) variants that were associated with biomarker levels at P < 1 × 10^−5^ and were located more than 500 kb away from any known index variant for the respective biomarker. Using data from up to 25,897 samples from 4 independent studies (Supplementary Table S1), five loci provided evidence of replication and reached P < 3.6 × 10^−9^ for the combined analysis of discovery and replication data (Table 3).

**Table 3:** Association results of replicating novel signals.

biomarker	rs-id	function	nearest gene	cytoband	EA/NEA	discovery						replication			combined	
						EAF	beta (SE), p-value	N	r^2^	imputed	EAF	beta (SE), p-value	N	EAF	beta (SE), p-value	N
Alanine Transaminase	rs28929474	missense	*SERPINA1*	14q32	T/C	0.02	0.04 (0.01), 2.61 × 10^−6^	9731	1.00	no	0.02	0.04 (0.01), 1.47 × 10^−5^	9881	0.02	0.04 (0.01), 1.72 × 10^−10^	19612
Dihydroepiandrosterone Sulphate	rs78900934	upstream gene	*PPIAP7*	1p21	A/C	0.31	0.05 (0.01), 7.95 × 10^−8^	9722	1.00	yes	0.31	0.08 (0.02), 4.32 × 10^−6^	3630	0.31	0.05 (0.01), 5.88 × 10^−12^	13352
Dihydroepiandrosterone Sulphate	rs2911280	intron	*CMIP*	16q23	A/G	0.08	0.09 (0.02), 2.25 × 10^−8^	9722	0.97	yes	0.07	0.08 (0.03), 8.63 × 10^−3^	3630	0.08	0.09 (0.01), 5.97 × 10^−10^	13352
Glycated haemoglobin	rs12819124	intron	*RP1-228P16.4*	12q13	A/C	0.47	−0.04 (0.01), 5.94 × 10^−8^	9436	0.99	yes	0.47	−0.02 (0.01), 1.12 × 10^−3^	7970	0.47	−0.03 (0.01), 4.20 × 10^−9^	17406
Glycated haemoglobin	rs761772	non-coding exonic	*TMC6*	17q25	C/T	0.13	0.06 (0.01), 5.94 × 10^−8^	9436	0.92	yes	0.12	0.03 (0.01), 3.83 × 10^−3^	5190	0.12	0.05 (0.01), 4.86 × 10^−9^	14626

function: variant functional consequence; nearest gene: gene nearest to lead variant with 500Kb from either side; chr: chromosome; EA/NEA: effect allele/non-effect allele; EAF; effect allele frequency; beta(SE), p-value: effect size (standard error) and p-value; N: total number of individuals analysed for this variant; r^2^: imputation accuracy.

**Figure 3:**
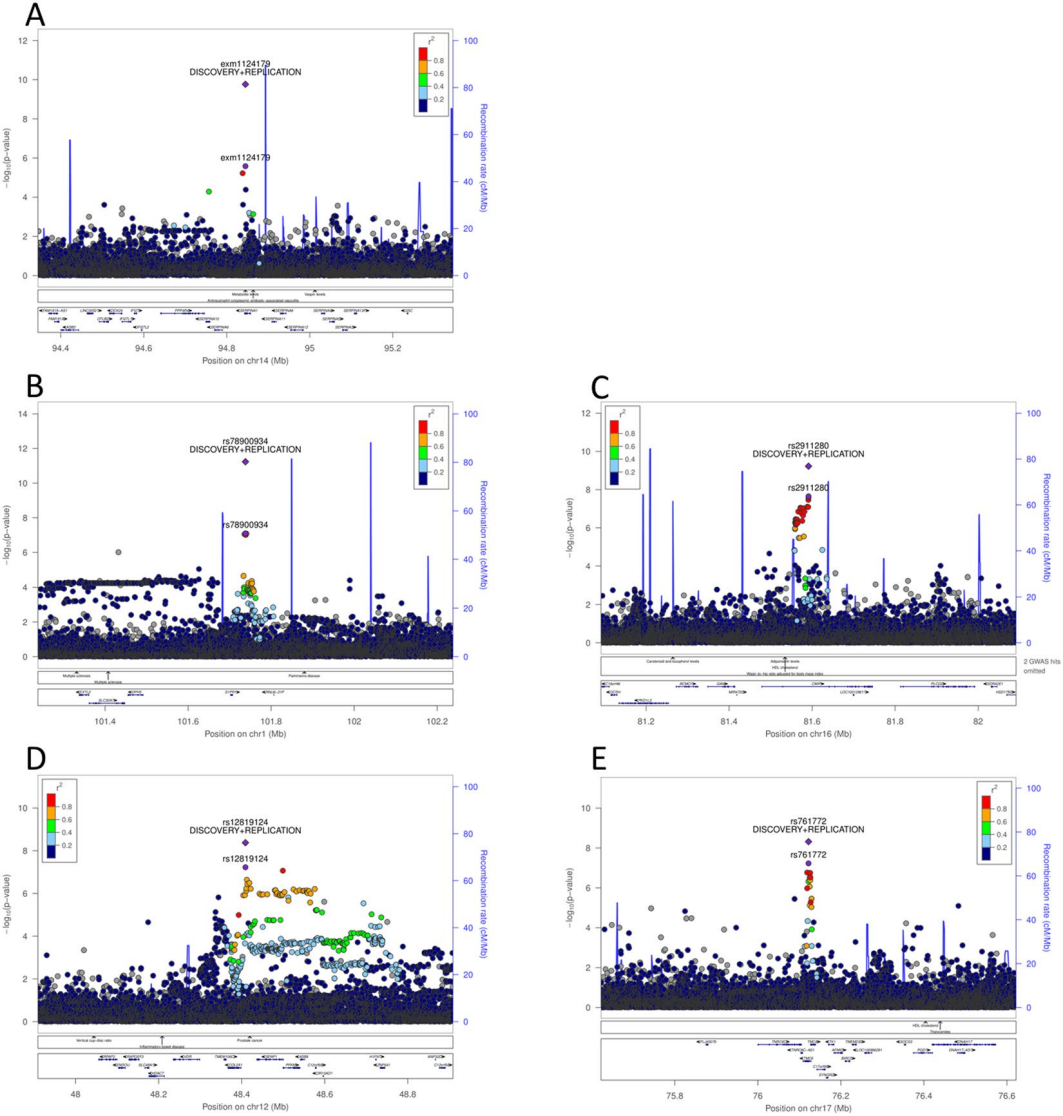
Regional association plots of novel genome-wide significant loci. Panel A–E : Regional association plots for replicating lead variants for alanine transaminase (**A**), DHEAS (**B**,**C**), HbA1c (**D**,**E**) respectively. Pairwise LD (r2) with the index variant is indicated following a color-coded scale. Both the p-values for the discovery as well as the combined discovery + replication are plotted for the index variant, results for all other variants were based on discovery-only data.

**Figure 4:**
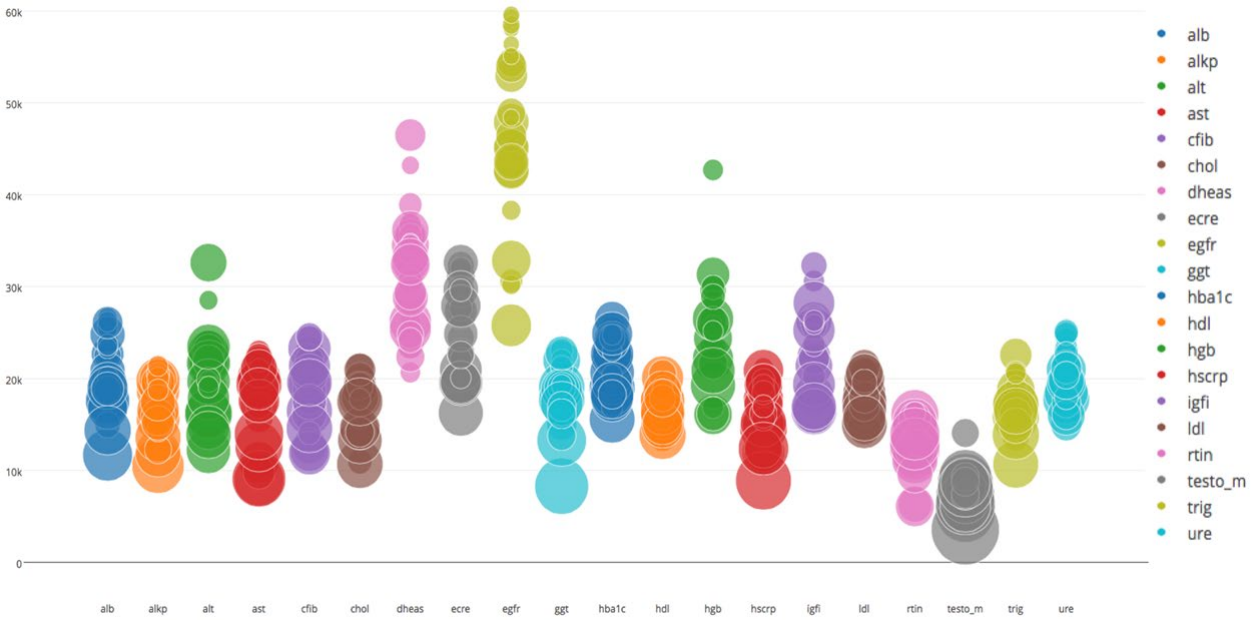
Power calculations. Power calculations for individual variants selected for replication per trait, number of samples needed to reach 80% power to reach genome-wide significance (P<3.56 × 10^−9^, two-sided). The size of the circles represents the relative effect size (standardised) compared amongst all traits.

rs28929474 at 14q32 (WEAF = 2%, beta[SE] = 0.04[0.01], P = 1.7 × 10^−10^), a low-frequency variant associated with alanine transaminase (ALT), resides in the serpin family A member 1 (*SERPINA1*) gene (Figs 3 A and 4). *SERPINA1* encodes alpha-1-antitrypsin (AAT), which is a serine protease inhibitor produced in the liver^11^. Low levels of this protein are the hallmark of a genetic disorder called alpha-1 antitrypsin deficiency (A1AD), which leads to liver disease^12^.

We identified two novel replicating associations for DHEAS (Fig. 3B,C). DHEAS is the sulphated form of DHEA, a precursor to major sex-hormones such as testosterone and oestrogen, and is synthesized in the adrenal glands. It is an important marker of adrenal gland function. rs78900934 at chromosome 1p21 (WEAF = 30.9%, beta[SE] = 0.05[0.01], P = 5.9 × 10^−12^) is located 1 kb upstream of a pseudogene, peptidylprolyl isomerase A pseudogene 7 (*PPIAP*7). This gene shows a high degree of similarity to cyclophilin A (*PPIA*), the product of which is involved in a number of biological processes including signal transduction^13^, inflammation^14^ and apoptosis^15^. At the second novel locus associated with DHEAS the index variant, rs2911280 at 16q13 (WEAF = 7.5%, beta[SE] = 0.09[0.01], P = 6.0 × 10^−10^), is located in an intron of the gene encoding c-Maf inducing protein (*CMIP*), thought to play a role in the T-cell signalling pathway.^16^

Two novel replicating associations with HbA1c levels were identified (Fig. 3D,E). HbA1c represents the three-month average plasma glucose concentration and is used to diagnose as well as manage type 2 diabetes. The index variant at 12q13, rs12819124 (WEAF = 46.7%, beta[SE] = −0.03[0.01], P = 4.2 × 10^−9^) lies in an intron of *RP1-228P16.4*, a long non-coding RNA. The index variant of the second novel locus, rs761772 at 17q25 (WEAF = 12.4%, beta[SE] = 0.05[0.01], P = 4.9 × 10^−9^), lies within a non-coding exon in the transmembrane channel-like 6 (*TMC6*) gene and has been shown to affect the expression of *TMC6*, as well as *TNRC6C antisense RNA 1* (*TNRC6C-AS*1) and transmembrane channel like 8 (*TMC8*), in cardiac, thyroid, and vascular tissue, as well as whole blood in the GTEx database^17^.

## Discussion

We identify five new biomarker loci, across common and low frequency variants, associated with DHEAS, HbA1c and ALT. We demonstrate polygenic heritability of the majority of biomarkers included in this study and observe large differences in their polygenic component. To our knowledge this is the first report of SNP-based heritability estimates for DHEAS, insulin-like growth factor 1, testosterone and urea. The large sample set with homogeneous biomarker measurements afforded by UKHLS enables reliable estimation for this population. We also identify genetic correlations between several of the biomarkers. Genetic correlation between two traits is an indicator of shared genetic factors and consequently genome-wide pleiotropy. The patterns of heritability and genetic correlations we observe for lipid biomarkers are consistent with previous reports in independent samples^18^. For total and LDL cholesterol, the SNP-based heritability is less than 10% whilst for HDL it is higher at 23.2%. All these estimates represent a lower bound for the narrow sense heritability. Our estimate of the negative genetic correlation between levels of HDL-cholesterol with triglycerides of rg = −0.67 is similar to the estimate derived from an independent study (rg = −0.61)^19^. High levels of triglycerides are mechanistically related to low levels of HDL^20, 21^, which could explain the reverse influence of the shared genetic factors on the biomarkers. We show for the first time that polygenic factors for triglyceride are also negatively correlated with DHEAS. There is a statistically significant genetic correlation between CRP and fibrinogen levels, which could be due to shared inflammation pathways. Finally, the genetic correlation we observe between creatinine and urea is a previously unreported and highly biologically plausible finding as both markers are increased in blood when glomerular filtration rate declines, reflecting impaired kidney function. Characterising the genetic architecture of health-related biomarkers in this way is informative with respect to their biology as well as the design of future association studies. While each known locus individually explains only a small proportion of the variance in biomarker levels, these analyses demonstrate that the joint effect of many variants can be much larger.

We examined less-well studied health-related biomarkers in addition to routine blood measures used in clinical practice. This made it possible to identify novel associations of common and low frequency variants with DHEAS, HbA1c and ALT. These associations could provide novel biological insights. rs2911280, which we found to be associated with DHEAS, is located in an intron of the gene encoding c-Maf inducing protein (*CMIP*). *CMIP* is a highly pleiotropic gene, and is associated with several metabolic traits such as adiponectin and HDL cholesterol levels. Cholesterol is a precursor of DHEA in its synthesis process^22^.

rs28929474 at 14q32 is associated with levels of ALT, which is used in clinical practice to assess liver damage. This variant is located in *SERPINA1*, encoding the serine protease inhibitor alpha-1-antitrypsin (AAT), which is largely produced in the liver. Associations of variants in this gene were previously found for cortisol^23^ and height^24^. Mutations of this gene can cause alpha-1 antitrypsin deficiency (A1AD) which can lead to an accumulation of aberrant proteins in hepatocytes causing liver damage^25^. This in turn may elevate levels of ALT, warranting future assessment of the association between this signal and liver-related clinical endpoints.

We identify two novel associations with HbA1c levels. In a lookup using published data from an independent large-scale meta-analysis by the MAGIC consortium^26^, rs12819124 was associated with HbA1c levels with P = 1.8 × 10^−6^. The direction of effect was consistent with our findings. rs12819124 was also nominally associated with risk of type 2 diabetes at P = 0.025 using data from the DIAGRAM study^27^. Moreover, association results from published cohorts suggest a possible pleiotropic association with mental disorders and wellbeing (P = 9.0 × 10^−6^ for bipolar disorder and schizophrenia^28^, P = 6.4 × 10^−5^ for subjective wellbeing^29^). No HbA1c association results were available for rs761772 in MAGIC. For a proxy SNP, rs429216 (r^2^ = 0.75), the p-value for the association with HbA1c was in the same direction and reached P = 2.7 × 10^−3^.

The UKHLS sample size is modest compared to some of the previous large-scale GWAS meta-analysis efforts (e.g., >45,000 individuals for HbA1c levels^26^). The relative gain in power leading to novel locus identification in this study can be attributed to several factors. Two of the newly reported signals have relatively low allele frequency (2% and 7.5%, respectively). These were captured here through use of the Illumina HumanCoreExome array and imputation to a comprehensive reference panel consisting of 1000 Genomes combined with the UK10K haplotypes^30^. A further power advantage was afforded by the homogeneous measurement of biomarkers in UKHLS and in two of the replication studies. Each biomarker was measured using the same assay for each sample, and processed on the same machine, avoiding loss of information due to diverse biomarker assays with different sensitivity, dynamic range and detection limit, potentially leading to power reductions^31^.

Larger-scale homogeneous studies and synthesis in massive-scale meta-analyses will help further elucidate the genetic architecture of medically-relevant biomarker traits. Insights into the genetic determinants of population variation in biomarker levels can help us to understand basic processes involved in maintaining health.

## Methods

### Ethics

Participants gave informed written consent for their blood to be taken and stored for future scientific analysis. The UKHLS has been approved by the University of Essex Ethics Committee and the nurse data collection by the National Research Ethics Service (10/H0604/2).

### Study population

The United Kingdom Household Longitudinal Study, also known as *Understanding Society* (https://www.understandingsociety.ac.uk) is a longitudinal panel survey of 40,000 UK households from England, Scotland, Wales and Northern Ireland). Participants are surveyed annually since 2009 and contribute information relating to their socioeconomic circumstances, attitudes, and behaviours via a computer assisted interview. As recruitment was household based, the study contains related individuals. The study includes phenotypical data for a representative sample of participants for a wide range of social and economic indicators as well as a biological sample collection encompassing biometric, physiological, biochemical, and haematological measurements and self-reported medical history and medication use (https://www.understandingsociety.ac.uk/d/100/7251_User_Guide_Health_Assmt_w2_w3.pdf?1392855567). For each participant non-fasting blood samples were collected through venepuncture, were centrifuged to separate plasma and serum, aliquoted and frozen at −80 °C. DNA has been extracted and stored for genetic analyses.

For replication, data were available for 5533 individuals from ELSA^32^, 9888 from Fenland^33^ (Supplemental Table 1), 7621 from HRS (http://hrsonline.isr.umich.edu)^34^, 2859 from NCDS^35^. These studies have been described in detail elsewhere. Sample collection were carried out consistently and analysed by the same laboratories for UKHLS, ELSA and NCDS.

### Biomarker measurements

In total, biomarker data were successfully obtained from 13,107 eligible individuals who gave consent to give blood samples to be stored for future analysis (https://www.understandingsociety.ac.uk/d/154/7251-UnderstandingSociety-Biomarker-UserGuide-2014.pdf?1418057881). All biomarkers were measured from serum (non-fasting), using a variety of suitable assays, and the majority analysed on a single Roche P module analyser^36^. On each machine Internal Quality Controls (IQC) were at regular intervals per day. External Quality Assurance (EQA) systems were in place to monitor all tests.

### Phenotype transformations and exclusions

The measurements for biomarkers used in the association analyses were prepared according to protocols from the largest genetic association study published for each specific trait at the time when analyses commenced, details for which are available in Supplementary Table S2.

### Genotyping

In total, 10,484 UKHLS samples have been typed using the Illumina Infinium HumanCoreExome BeadChip Kit® (12v1-0). This array contains a set of >250,000 highly informative genome-wide tagging single nucleotide polymorphisms as well as a panel of functional (protein-altering) exonic markers, including a large proportion of low-frequency (MAF 1–5%) and rare (MAF <1%) variants. Genotype calling was performed with the gencall algorithm using GenomeStudio (Illumina Inc.). For quality control (QC) we excluded individuals based on the following criteria: sample call rate <98%, autosomal heterozygosity outliers (>3SD), gender mismatches, duplicates as established by identity by descent (IBD) analysis (PI_HAT > 0.9). Individuals with non-European ancestry were also excluded. For this we estimated the genomic kinship between all pairs of individuals along with 1000 Genomes Project data. These were converted to distances and subjected to multidimensional scaling. Prior to variant QC, we first mapped all 538,448 variants to the human reference genome build 37. Variants with Hardy-Weinberg equilibrium p-value < 1 × 10^−4^, call rate below 98% or poor genotype clustering values (<0.4) were excluded, leaving 525,314 variants passing QC. For typed variants in our GWAS analyses that were brought forward for replication we inspected cluster plots manually using Scattershot 0.75 beta (Supplementary Fig. S1). All QC procedures were carried out using PLINK (v1.07) and R.

### Imputation

We imputed our genotype data using a combined reference panel consisting of 7,562 haplotypes from the UK10K project and 2,184 haplotypes from 1000 Genomes phase 3. Details regarding the creation of this combined imputation panel are described elsewhere^37^. Prior to imputation, we first pre-phased using SHAPEIT (v2.r). Data were then imputed using IMPUTE2 (v2.3.0), resulting in an initial set of 38,310,212 variants. Variants with an IMPUTE info score <0.4, and variants with a Hardy-Weinberg p-value < 1 × 10^−4^ were excluded, leaving 26,851,013 variants for analysis.

### Data availability

The UKHLS EGA accession number is EGAD00010000918. ELSA EGA accession number is EGAC00001000270. NCDS accession number is EGAC00000000001. HRS is available through dbGAP, Study Accession number phs000428.v1.p1. Genotype-phenotype data access for UKHLS, ELSA and NCDS is available by application to Metadac (www.metadac.ac.uk).

### Statistical analysesh

#### Heritability analyses and genetic correlations

The proportion of trait variance explained by the genotyped and imputed variants was estimated using the GREML method as implemented in the GCTA software^38, 39^ (v1.26). We included all variants with minor allele frequency (MAF) > 0.01. We excluded variants with imputation accuracy less than 0.4. We computed the genetic relationship matrix (GRM) for each autosome and then used GCTA to combine them into one matrix. We excluded relatives from the estimation by filtering based on the GRM using a threshold of 0.1 after inspecting the distribution. This led to the exclusion of 672 individuals for this analysis. We also performed bivariate REML analysis in order to estimate genetic correlations between different biomarkers^40^. We applied a Bonferroni adjusted significance threshold using the effective number of traits for the heritability analyses and using the number of pairs based on the effective number of traits for the genetic correlation analyses.

#### Association analyses

The association analyses were carried out using a multivariate linear mixed model to account for relatedness as implemented in GEMMA (v0.95). QQ plots and genomic inflation factors, as well as Manhattan plots for traits where we identified novel associations are displayed in Supplementary Fig. S2. Replication analyses were carried out in R and PLINK, following the same trait preparation protocols as used in the discovery stage. The association summary statistics from the replication analyses, as well as the combined discovery and replication stage were meta-analysed using a fixed-effects inverse variance weighted approach implemented in METAL (v2011-03-25). We calculated an adjusted genome-wide significance threshold, for the effective number of traits, as several of our biomarkers have correlated levels. The effective number of traits was derived by computing the eigenvalues for the correlation matrix of the 20 biomarkers (effective number: 14.05). The routinely used GWAS threshold of p < 5 × 10^−8^ was then adjusted for this using the Bonferroni approach: 5 × 10^−8^/14.05 = 3.56 × 10^−9^.

#### Power calculations

We carried out power calculations using Quanto (v1.2.4), for discrete per-variant frequency and (standardised) effect sizes combinations, representative of variants identified in the discovery. Per-trait and per selected variant power analyses showed that we would minimally need 5,000 to 15,000 samples to replicate our variants with P < 3.56 × 10^−9^, two-sided, for testosterone levels, whereas the largest replication sample of 25,000 to 60,000 would be needed for eGFR (Fig. 4).

#### Selection of replication SNPs, and criteria for novel loci

For replication we selected independent SNPs (LD r2 < 0.1), with MAF > 0.01 and a discovery p-value of P < 1 × 10^−5^ and at least > 500 Kb away from the nearest known reported index SNP for a given trait. We also took forward independent rare variants with a MAF ≤ 0.01 that were typed and reached P < 1 × 10^−5^, regardless whether they represented known associations for a given trait. Known index SNPs for all biomarkers analysed in this study were obtained through the GWAS catalog^41^ (accessed August 4, 2016) > , supplemented by manual searches in PubMed.

#### Annotation

For annotation of our lead variants we used an in-house annotation script that automatically retrieves variant annotations from ENSEMBL^42^, including variant function, the nearest gene IDs within < 500Kb from a given variant, transcript and protein IDs for these genes, as well as conservation scores. It also calculates GWAVA^43^ scores for non-genic variants amongst other annotations.

All methods were performed in accordance with the relevant guidelines and regulations.

## Electronic supplementary material

Supplementary material
