# Supplementary material for: Genome-wide analysis of health-related biomarkers in the UK Household Longitudinal Study reveals novel associations
Source: Sci Rep. 2017 Sep 8;7:11008. doi: 10.1038/s41598-017-10812-1 (PMC5591265; doi:10.1038/s41598-017-10812-1)
Supplement: Supplementary file 1 — Supplementary material [file 41598_2017_10812_MOESM1_ESM.pdf]

Supplementary information

Genome-wide analysis of health-related biomarkers in the UK Household Longitudinal Study reveals novel associations

Bram P Prins[1¶], Karoline B Kuchenbaecker[1¶], Yanchun Bao[2], Melissa Smart[2], Delilah Zabaneh[3], Ghazaleh Fatemifar[4], Jian'an Luan[5], Nick J Wareham[5], Robert A Scott[5], John RB Perry[5], Claudia Langenberg[5], Michaela Benzeval[2], Meena Kumari[2], Eleftheria Zeggini[1]*

[1] Wellcome Trust Sanger Institute, Hinxton, UK

[2] Institute for Social and Economic Research, University of Essex, Wivenhoe Park, Colchester, Essex, UK

[3] MRC Social, Genetic & Developmental Psychiatry Centre, IoPPN, KCL, London

[4] Institute for Health Informatics, UCL and the Farr Institute of Health Informatics, London, , UK

[5] MRC Epidemiology Unit, University of Cambridge School of Clinical Medicine, Box 285 Institute of Metabolic Science, Cambridge Biomedical Campus, Cambridge, UK

Supplementary Table S1. Descriptive information about the UKHLS and Fenland cohorts.

	**STAGE**<br>**Cohort Acronym**	**DISCOVERY**<br>**UKHLS**	**Replication**<br>**Fenland**
STUDY INFORMATION	Cohort website (if available)	www.understandingsociety.ac.uk	http://www.mrc-epid.cam.ac.uk/research/studies/fenland/
	Ethnicity	European descent	European descent
	Country	United Kingdom	United Kingdom
	Study design	Population / household based, longitudinal	Population-based
	Primary GWAS or lookup (replication)	GWAS	lookup
Biomarker measurements	Biomarker assaying centre	Newcastle upon Tyne Hospitals NHS Foundations Trust (NUTH)	Clinical biochemistry, Cambridge University Hospitals NHS Foundation Trust
	Sample type (plasma/ serum etc.)	serum	Fasting fresh venous serum
	When possible, storage age of samples before measurements? (years)	up to 4 years	1 day
	When possible, date of sampling (years)	2010-2012	2005-2015
	Collection method (brief description of procedure)	Venous blood was centrifuged and serum frozen at -80C until measurement	Fasting fresh venous serum was directly sent to Biochemistry and measured within the same day
	Time between venisection and sample storage (-80°C) (hours)	62.4 hours (average 2.6 days)	
PARTICIPANTS : SELECTION AND INCLUSION IN ANALYSES	Inclusion/Exclusions criteria for participants (non-genetic)	All participants with biomarker measurements	participants are excluded from the Fenland study by their GP if they have been diagnosed with diabetes, have a terminal illness with a prognosis of less than one year, suffer from a psychotic illness, are pregnant or lactating, or are unable to walk unaided
	Total sample of Cohort [N All]	9965	12435
	Initial number of subjects with biomarker measurements initially included [N All - maximum (trait)]	9824 (albumin)	9896
	Age [Mean (SD) years] of included subjects [for N All Maximum]		48.3 (7.5)
	BMI of included subjects, (in kg / m2) [Mean (SD)] for N All Maximum]		26.9 (4.8)
	Percentage of female participants [Percentage from for N All Maximum included, listed at item 17]		53.11%
GENOTYPING	Genotyping centre	Wellcome Trust Sanger Institute	Cambridge Genomic Services, Department of Pathology, University of Cambridge, UK
	Genotyping Platform	Illumina HumanCoreExome v12.1	Affymetrix Axiom UKBiobank
	Genotyping calling algorithm	Gencall	Axiom GT1
SAMPLE QC	Heterozygosity [N individuals excluded / (>N SD from mean)]	3SD / 148	>0.19 and <0.21 calculated on SNPs with MAF > 1%, >0.004 and <0.0125 calculated on SNPs with MAF < 1%
	Sample call rate [N individuals excluded / filter detail]	33 / <98%	<95%
	Relatedness assessment and adjustment methods	pairwise IBD, adjust for relatedness in analyses using gemma v0.95 and kinship matrix, duplicates and MZ samples removed / 7	pairwise IBD, 1st & 2nd degree relativeness excluded

	Population stratification assessment and adjustment	MDS (PLINK), combining UKHLS with other ancestries from 1000 Genomes to remove ethnic outliers / 340	MDS (PLINK), exclude ancestry outliers (±10SD in any of the first 10 PCs)
	Other exclusions :	sex mismatches, Sequenom identity checks, exclusion samples that had mapping problems in the chain between survey office, fieldwork agency, storage facility and the genetics lab / 55, 3, 65	Channel contrast (DishQC <0.82), sex discrepancy, unusually high number of singleton genotypes, impossible IBD values
	Individuals for association analysis after sample genetic QC [N All]	9961	9896
	N of genotyped SNPs (before filtering) [N Total SNPs / N Autosomal SNPs]	535891 / 522709	
SNP QC	MAF [N Total SNPs excluded / filter detail]	NA / NA	excluded monomorphic
	HWE [N Total SNPs excluded / filter detail]	excluded if p≤10-4 / 4234	<10-6
	Call rate [N Total SNPs excluded / filter detail]	excluded if callrate <95% / 9128	<95%
	Other SNP QC filters applied after genotyping ? [N Total SNPs / filter detail]	Cluster separation score <0.4, X-chr nonPAR SNPs that have heterozygous haploid males / 1656, 966	Clusters pass Affymetrix SNPolisher standard tests and thresholds. MAF not significantly affected by plate (excludsion threshold p< 9.5e-8 and <20% difference in MAF), Y and MT chromosome removed; duplicates and unflippable SNPs removed
	N of genotyped SNPs after filtering [N Total SNPs / N Autosomal SNPs]	525314 / 510845	
IMPUTATION STATS	Imputation software	IMPUTE2	IMPUTE2
	Reference Panel: imputation backbone [Panel, NCBI Human genome build]	UK10K + 1000 Genomes, GRCh37 (b37) / hg19	1000G phase 3
	N SNPs used for imputation [N Total SNPs / N Autosomal SNPs]	351665 / 351665	718409 / 699990
	If any filters applied on imputed genotypes, please mention filter criteria and number of SNPs removed [filter detail]	pHWE < 1x10-4, IMPUTE info score < 0.4,	INFO<0.4, mornomorphic and singletons
DATA ANALYSIS	N of SNPs for analysis (after imputation and possible filtering) [N Total SNPs / N Autosomal SNPs]	26851013 / 26851013	332
	Software for analysis (name, version)	GEMMA v0.95, SNPTEST v2.5.1 (variant metrics only)	Stata
	Statistical analysis : model	Additive	Additive
	Statistical analysis : association testing method	multivariatelinear mixed model regression	Linear regression

Supplementary Table S2. Means, range and variable transformation details for biomarkers.

Phenotype	Abbreviation	Units	Mean	Minimum-maximum	Transformation	Exclusions based on distribution	Covariates used in regression	Transformation of residuals
Albumin	alb	g/dL	46.8	36-57	-	±4 SD	age, sex	-
Alkaline Phosphatase	alkp	IU/L	85.1	28-222	log10	±4 SD	age, sex	-
Alanine Transaminase	alt	IU/L	29.2	4-166	log10	±4 SD	age, sex	-
Aspartate Transaminase	ast	units/L	29.0	12.0-84.0	log	±4 SD	age, age2,sex	-
Fibrinogen	cfib	g/L	2.8	1.5-5.2	log	±3 SD	age,sex	-
Total Cholesterol	chol	mg/dL	209.3	81.1-359.1	-	±4 SD	age,age2, sex	invnt
LDL cholesterol	ldl	mg/dL	131.5	18.1-270.7	-	±4 SD	age,age2, sex	invnt
Dihydroepiandrosterone Sulphate	dheas	umol/L	3.6	0.4-25.3	log	±3 SD	age,sex	-
Creatinine	ecre	mg/dl	0.8	0.4-2.0	log	±4 SD	age, sex	-
Gamma Glutamyl Transferase	ggt	IU/L	33.9	7-246	log10	±4 SD	age, sex	-
Glycated haemoglobin	hba1c	%, NGSP	4.1	1.7-6.4	-	> 6.5% (otherwise diabetic)	age, sex	-
HDL cholesterol	hdl	mg/dL	58.7	15.8-122.8	-	±4 SD	age,age2, sex	invnt
Haemoglobin	hgb	g/dl	13.7	8.2-18.5	-	±4 SD	age,sex	-
C-Reactive Protein (high sensivity assay)	hscrp	mg/L	1.6	0.2-115.5	log	±4 SD	age,sex	-
Insulin-like growth factor 1	igfi	ng/mL	138.0	15.3-359.6	-	±4 SD	age, sex	-
Ferritin	rtin	ug/L	93.5	3.0-3044.2	log	±4 SD	age, sex	Z-score
Testosterone	testo	nmol/L	14.5	2.9-40.1	log	±4 SD	age	-
Triglycerides	trig	mg/dL	102.0	8.8-389.4	-	±4 SD	age,age2, sex	invnt
Urea	ure	mg/dl	36.1	12.7-99.4	log	±4 SD	age, sex, BMI	-

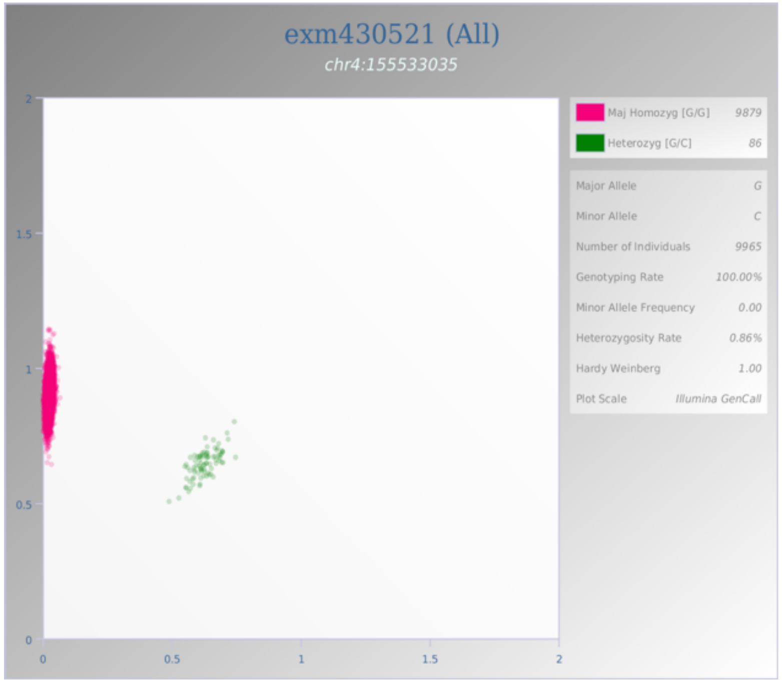

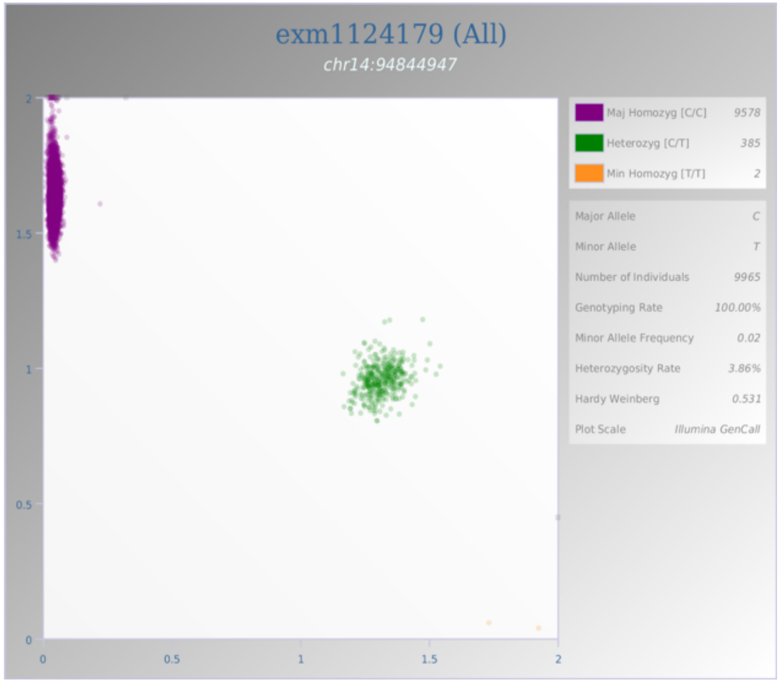

Supplementary Figure S1. Cluster plots for two variants amongst the novel association signals that were genotyped.

The subsequent pages show
Supplementary Figure S2. Manhattan and QQ-plots for biomarkers: albumin, alkaline phosphatase, alanine transaminase, aspartate transaminase, fibrinogen, total cholesterol, LDL cholesterol, dihydroepiandrosterone sulphate, creatinine, gamma glutamyl transferase, glycated haemoglobin, HDL cholesterol, haemoglobin, C-reactive protein, insulin-like growth factor 1, ferritin, testosterone, triglycerides, urea.

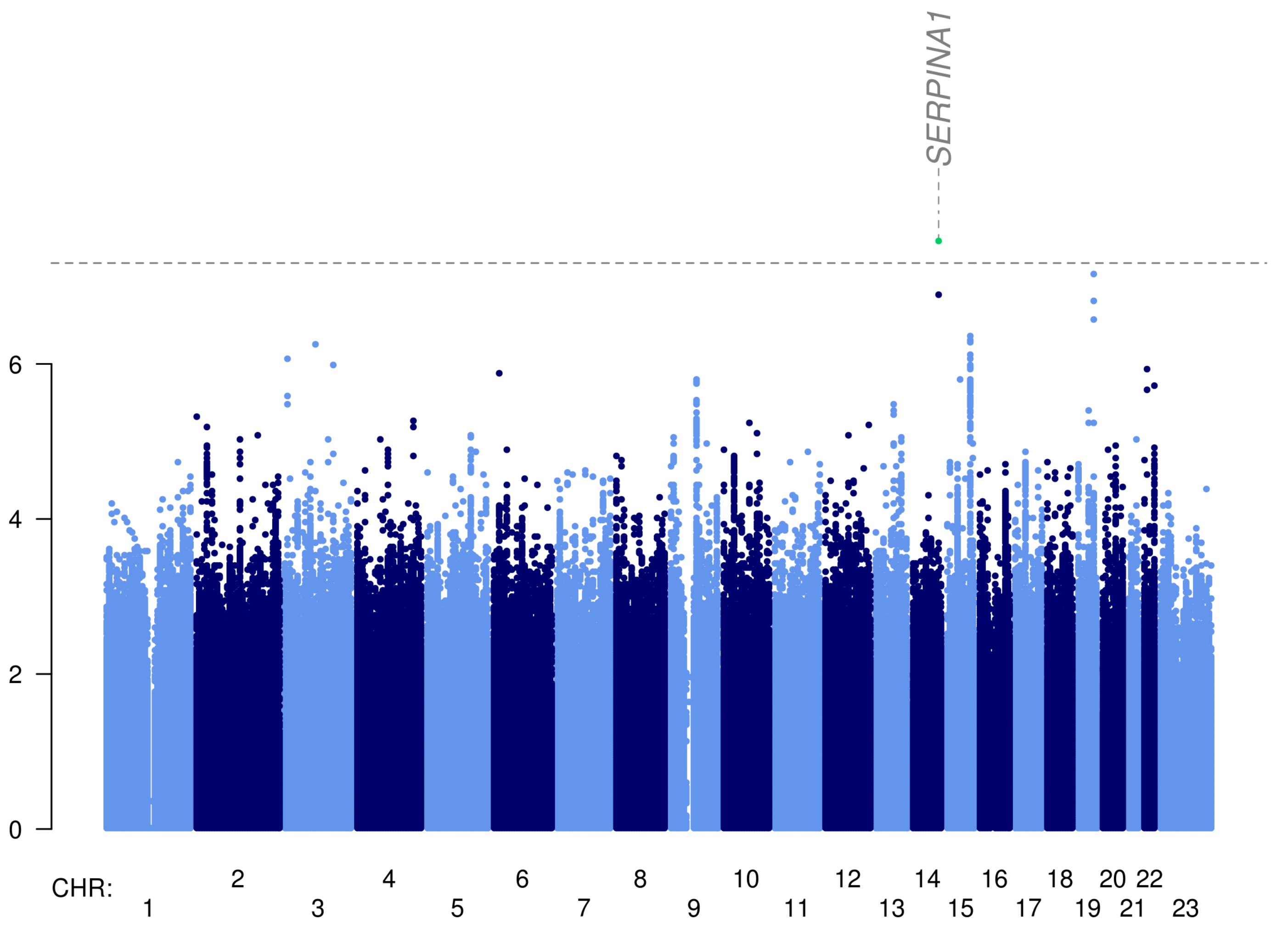
SERPINA1
6
4
2
0
CHR:
1 2 3 4 5 6 7 8 9 10 11 12 13 14 15 16 17 18 19 20 21 22 23

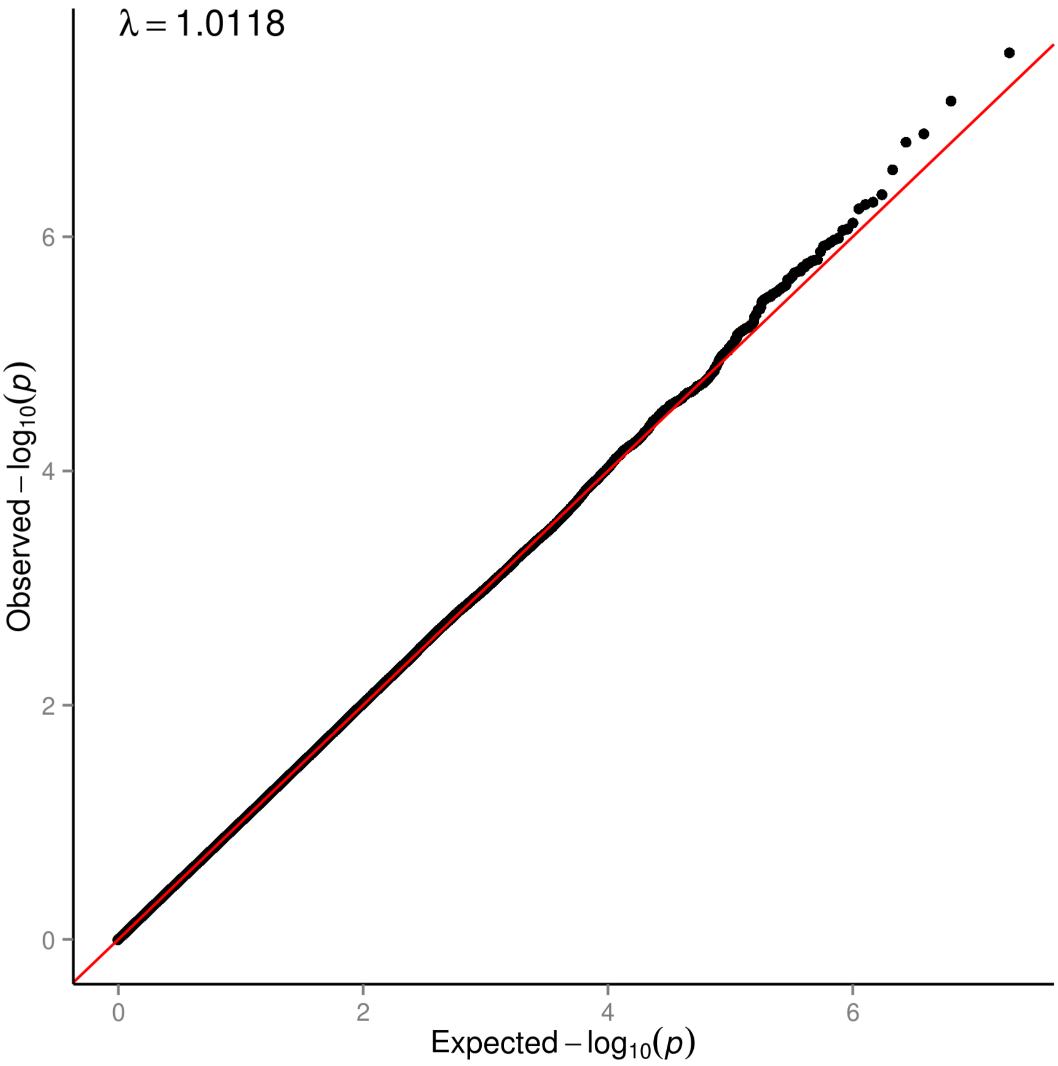

λ = 1.0118
Observed − log10(p)
Expected − log10(p)
0
2
4
6
0
2
4
6

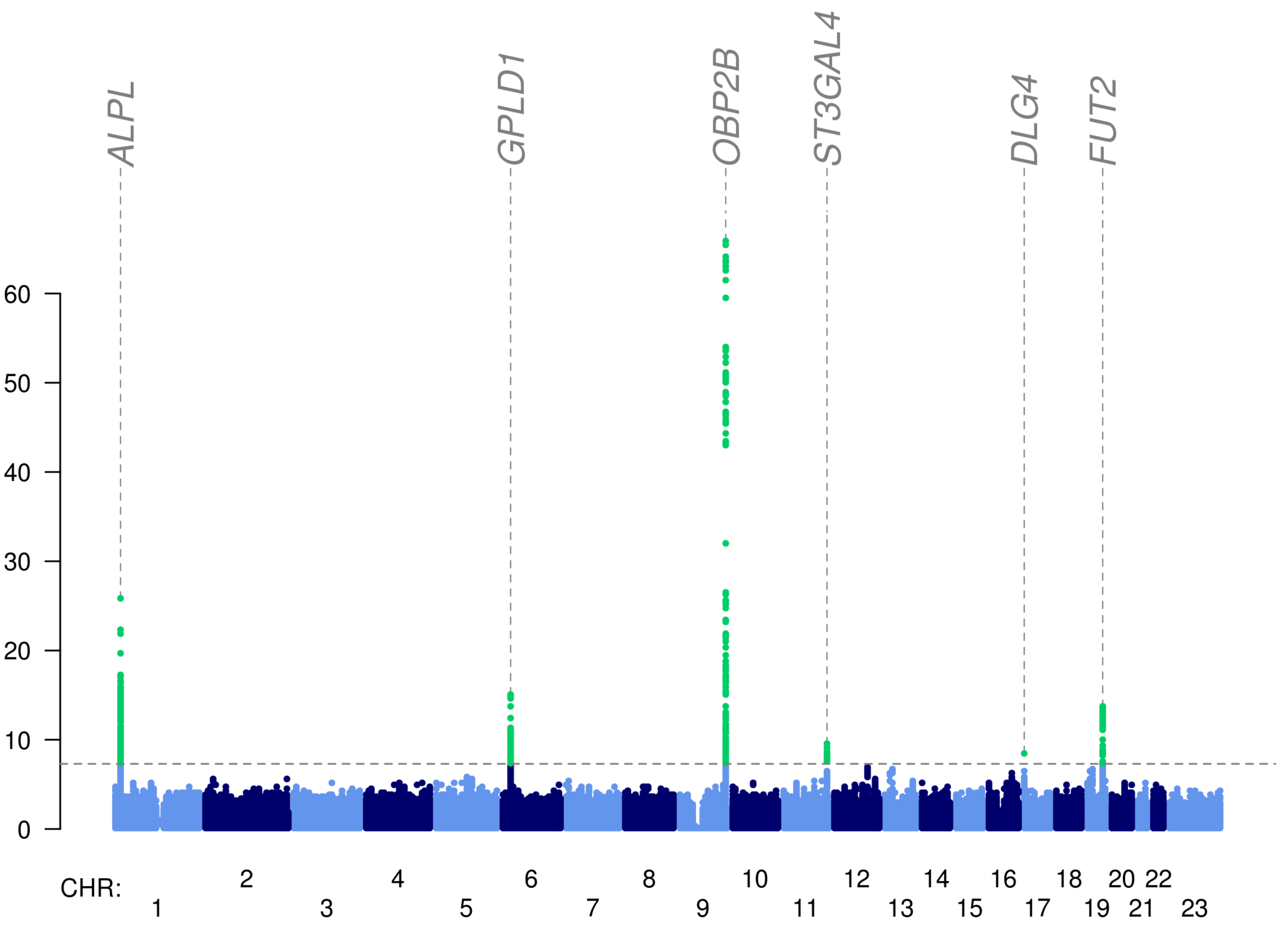
ALPL
GPLD1
OBP2B
ST3GAL4
DLG4
FUT2
60
50
40
30
20
10
0
CHR:
1 2 3 4 5 6 7 8 9 10 11 12 13 14 15 16 17 18 19 20 21 22 23

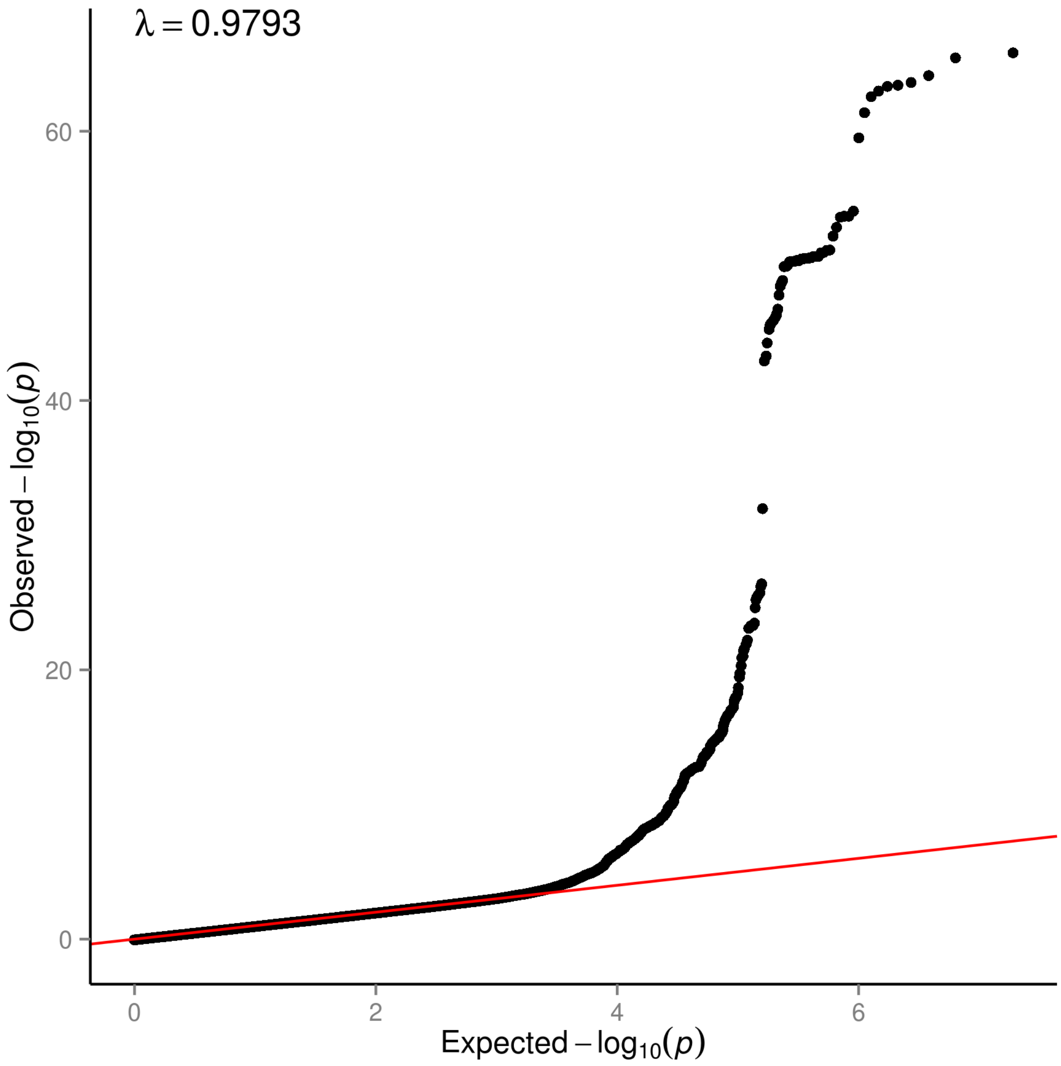

λ = 0.9793
Observed − log10(p)
Expected − log10(p)
0
20
40
60
0
2
4
6

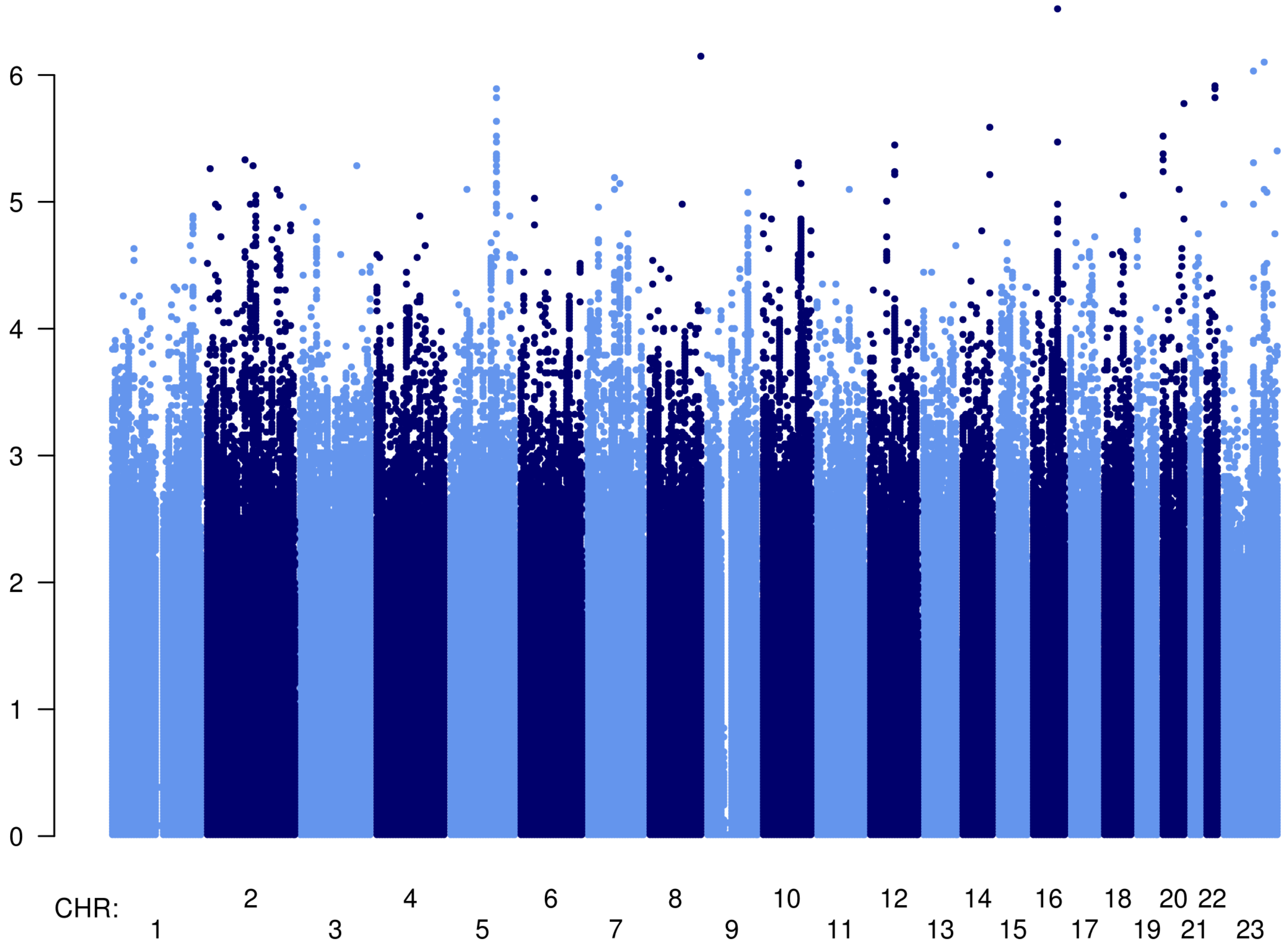

6
5
4
3
2
1
0
CHR:
2
4
6
8
10
12
14
16
18
20
22
1
3
5
7
9
11
13
15
17
19
21
23

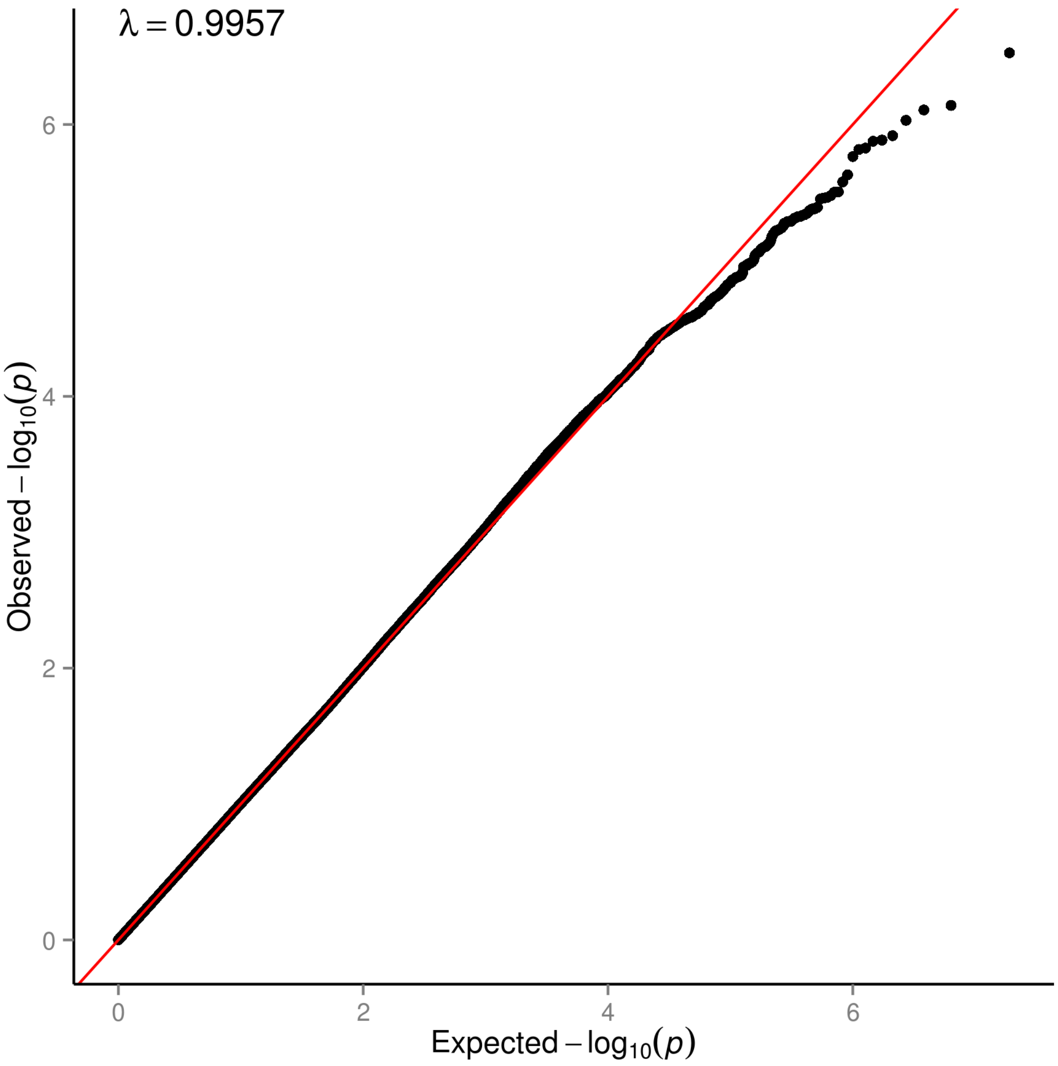
λ = 0.9957
Observed − log10(p)
Expected − log10(p)
0
2
4
6
0
2
4
6

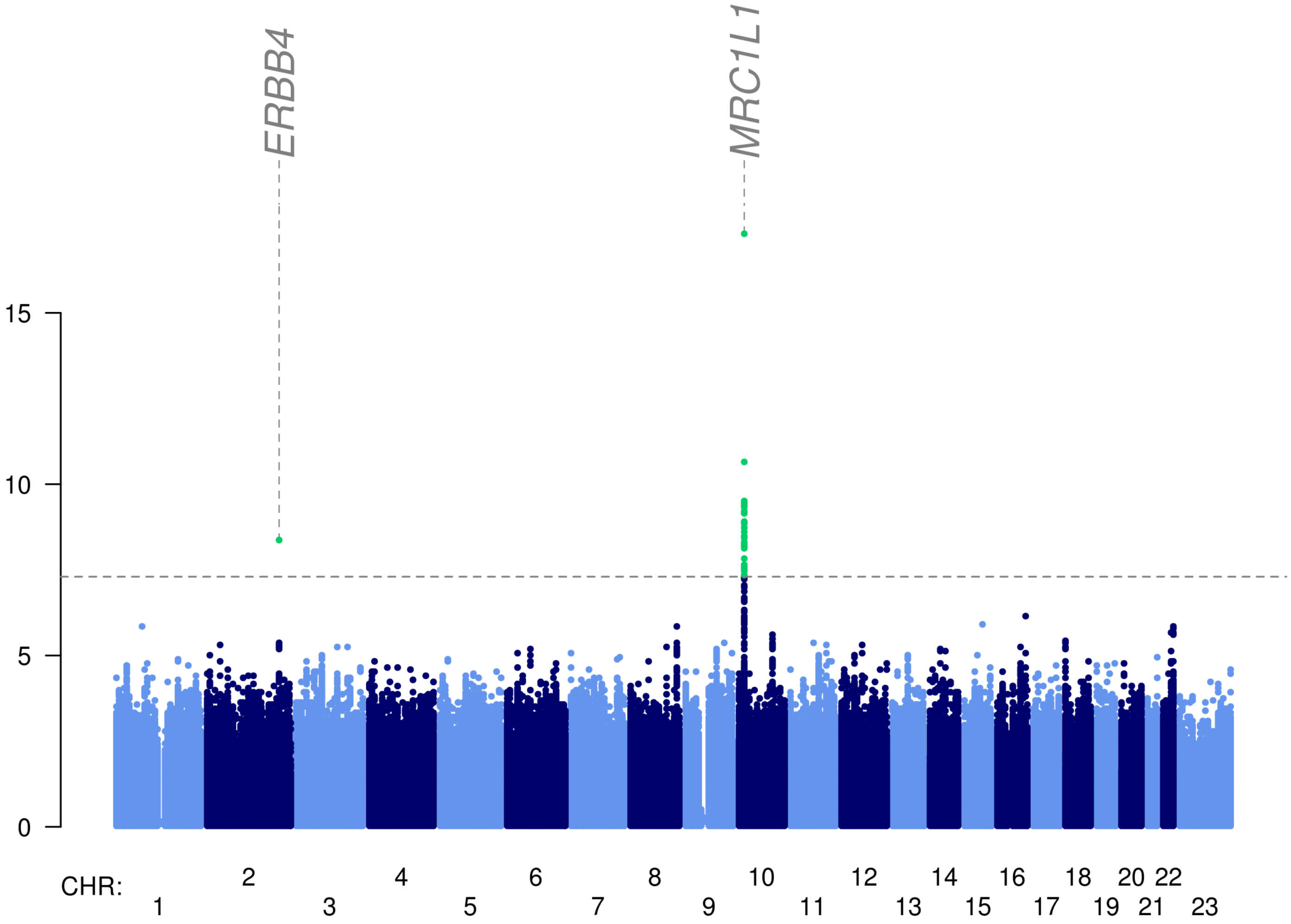
ERBB4
MRC1L1
15
10
5
0
CHR:
1 2 3 4 5 6 7 8 9 10 11 12 13 14 15 16 17 18 19 20 21 22 23

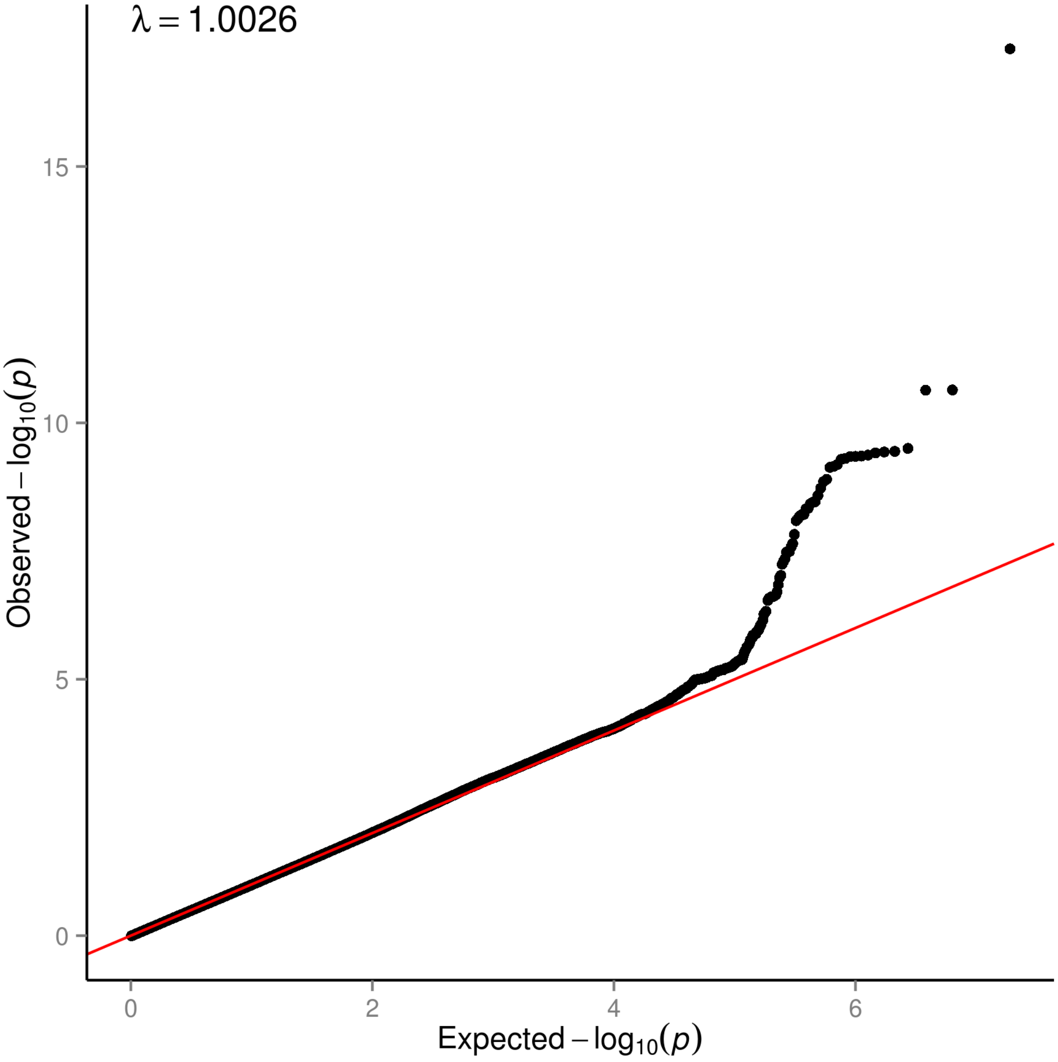

λ = 1.0026
15
10
5
0
Observed − log10(p)
0
2
4
6
Expected − log10(p)

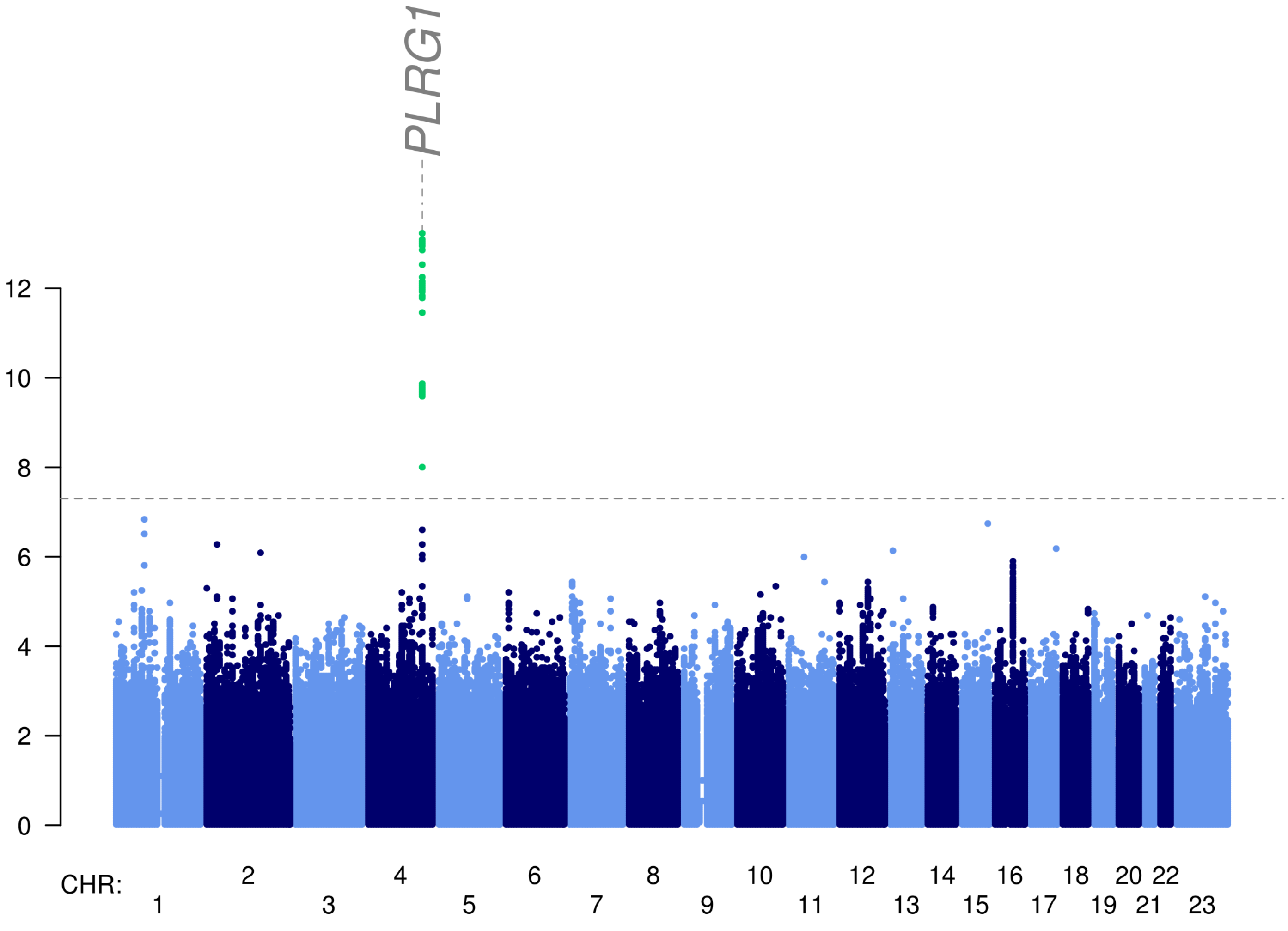

PLRG1
12
10
8
6
4
2
0
CHR:
1
2
3
4
5
6
7
8
9
10
11
12
13
14
15
16
17
18
19
20
21
22
23

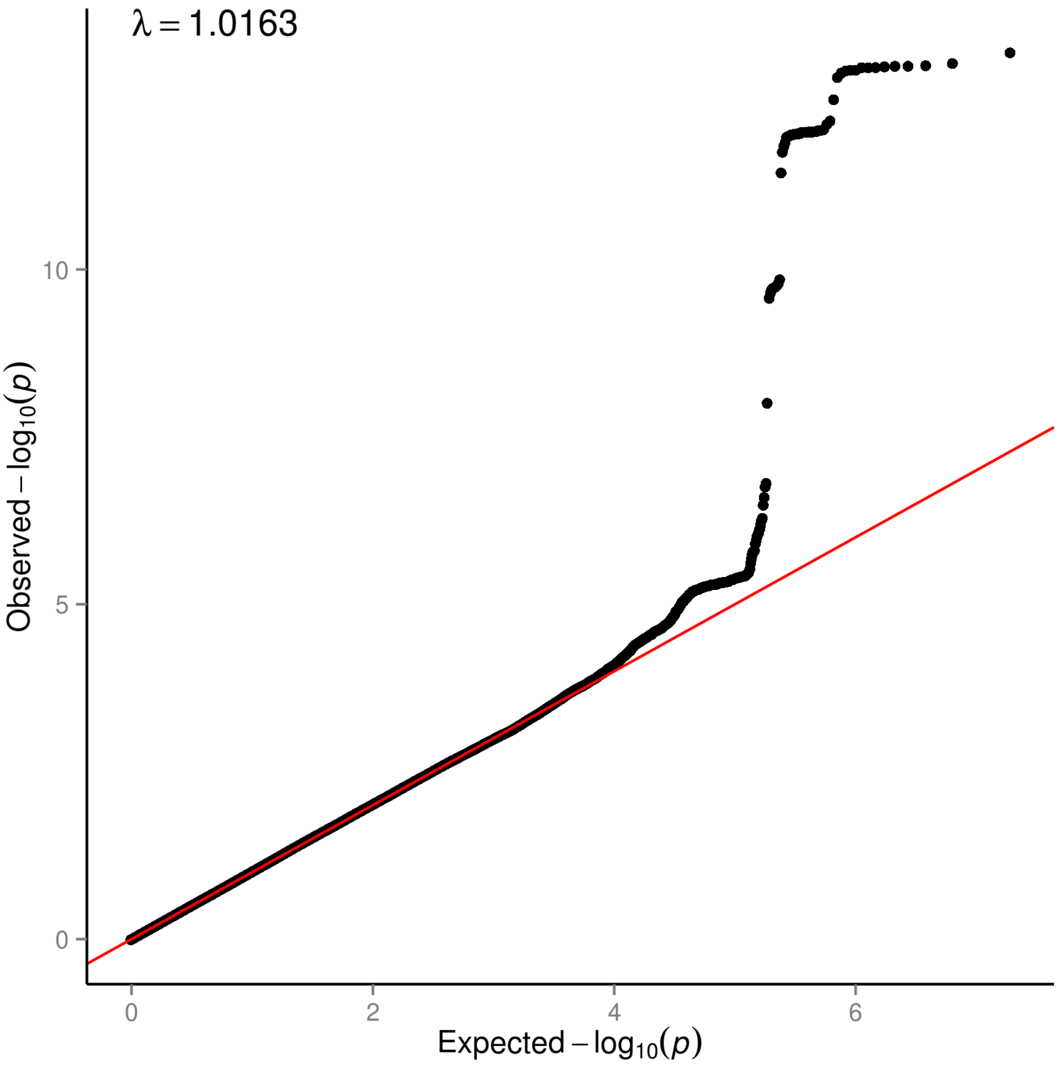
λ = 1.0163
Observed − log10(p)
Expected − log10(p)
0
5
10
0
2
4
6

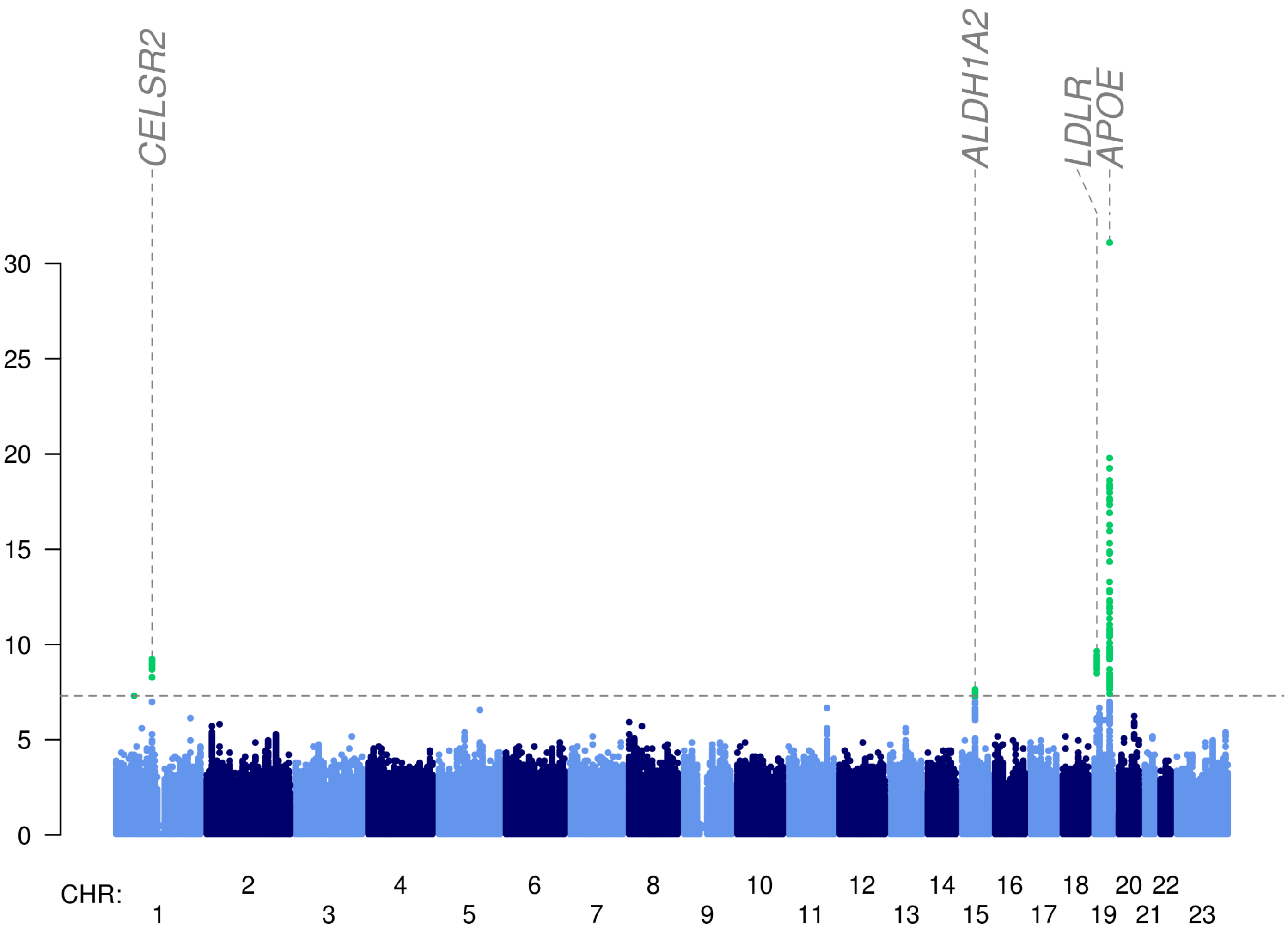
CELSR2
ALDH1A2
LDLR
APOE
30
25
20
15
10
5
0
CHR:
2
4
6
8
10
12
14
16
18
20
22
1
3
5
7
9
11
13
15
17
19
21
23

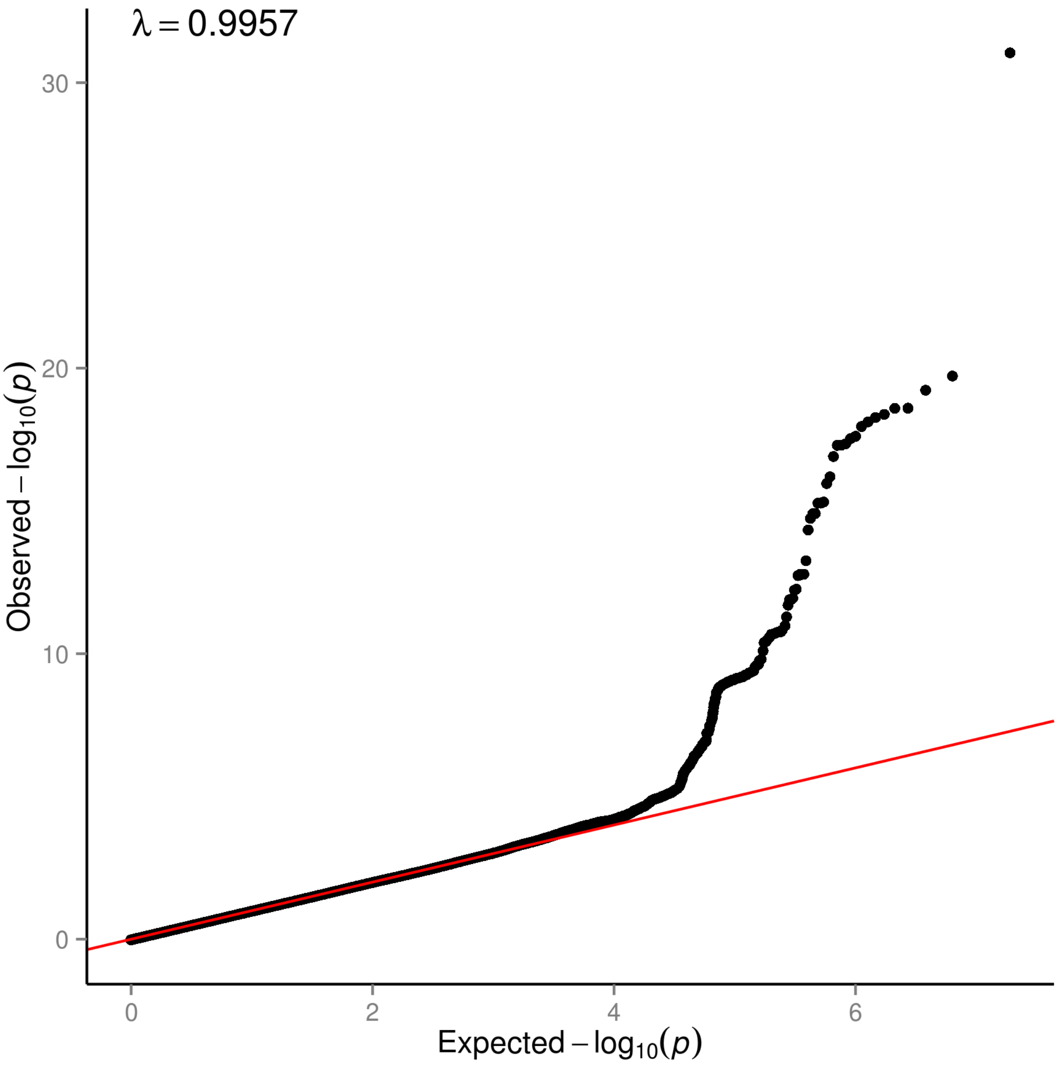

$\lambda = 0.9957$
30
20
10
0
Observed $-\log_{10}(p)$
0
2
4
6
Expected $-\log_{10}(p)$

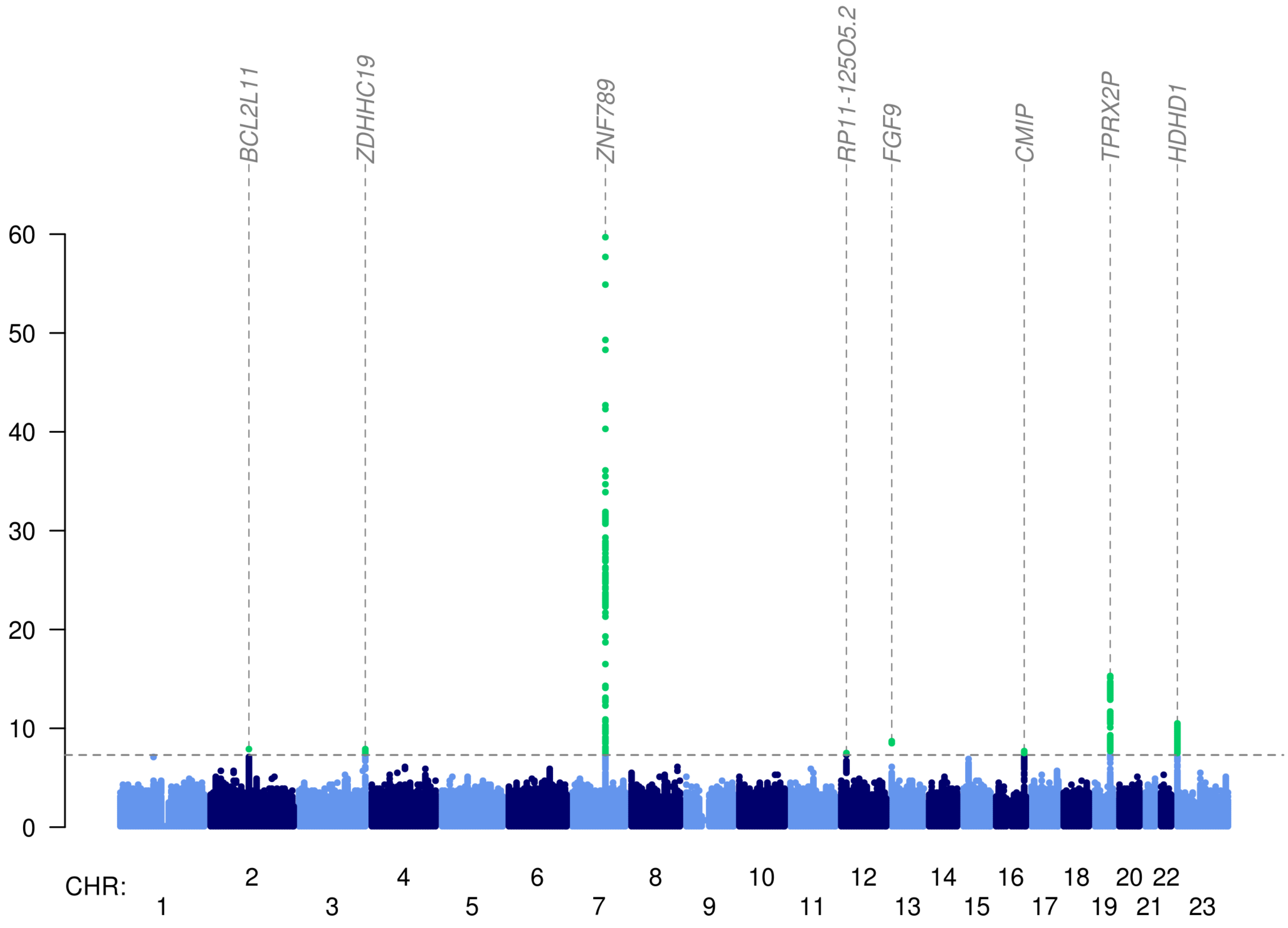

BCL2L11
ZDHHC19
ZNF789
RP11-125O5.2
FGF9
CMIP
TPRX2P
HDHD1
60
50
40
30
20
10
0
CHR:
1
2
3
4
5
6
7
8
9
10
11
12
13
14
15
16
17
18
19
20
21
22
23

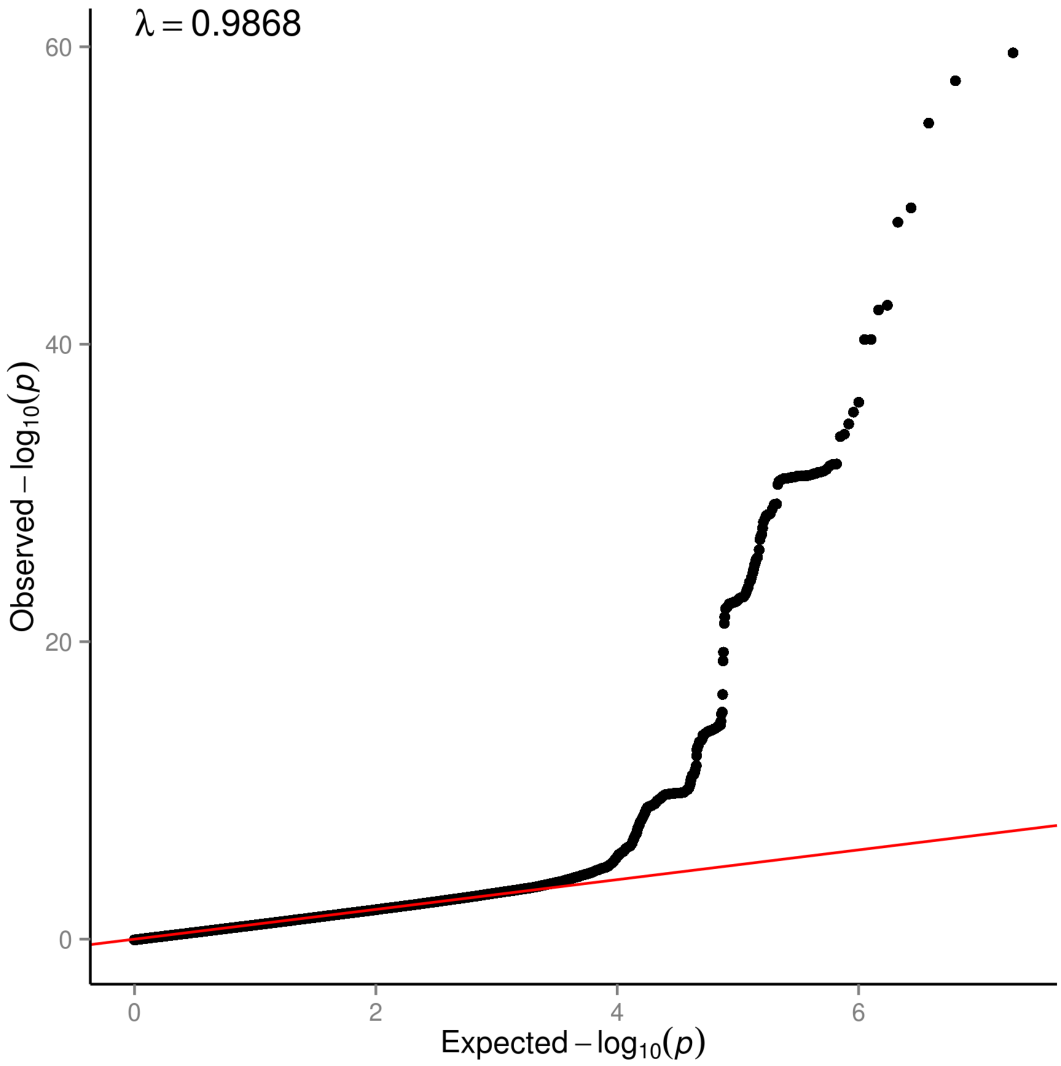

λ = 0.9868
60
40
20
0
0
2
4
6
Observed − log10(p)
Expected − log10(p)

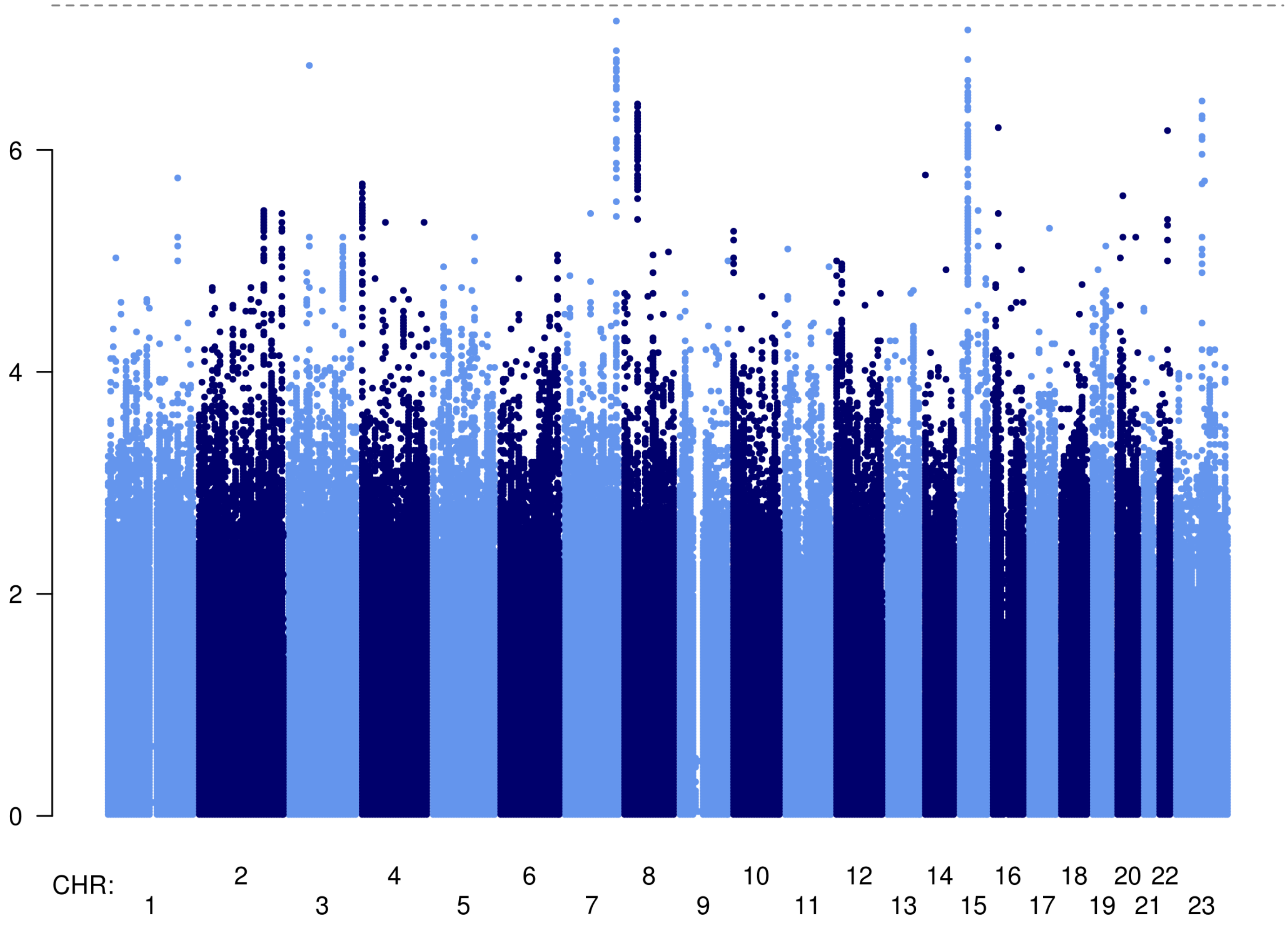

6
4
2
0
CHR:
2
4
6
8
10
12
14
16
18
20
22
1
3
5
7
9
11
13
15
17
19
21
23

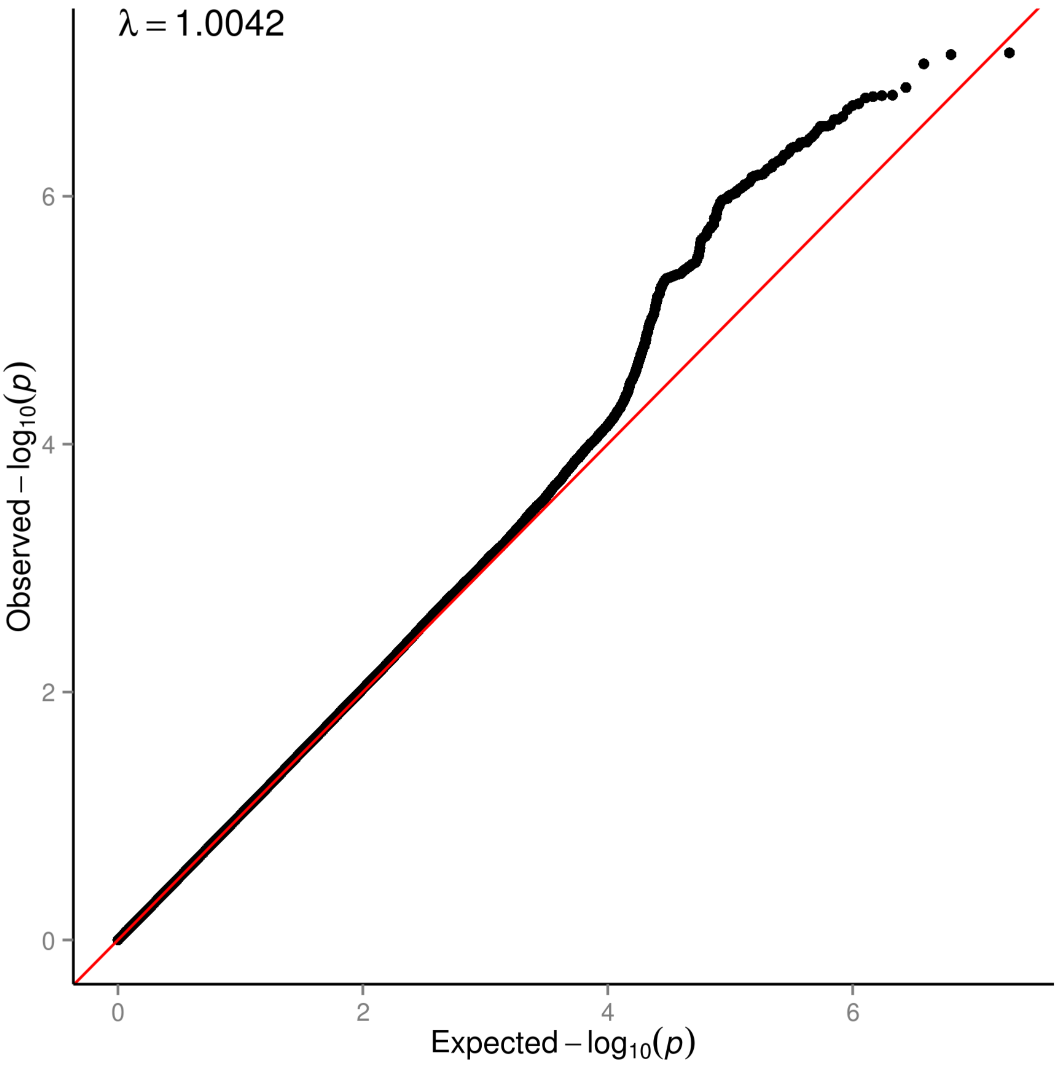

λ = 1.0042
Observed −log10(p)
Expected −log10(p)
0
2
4
6

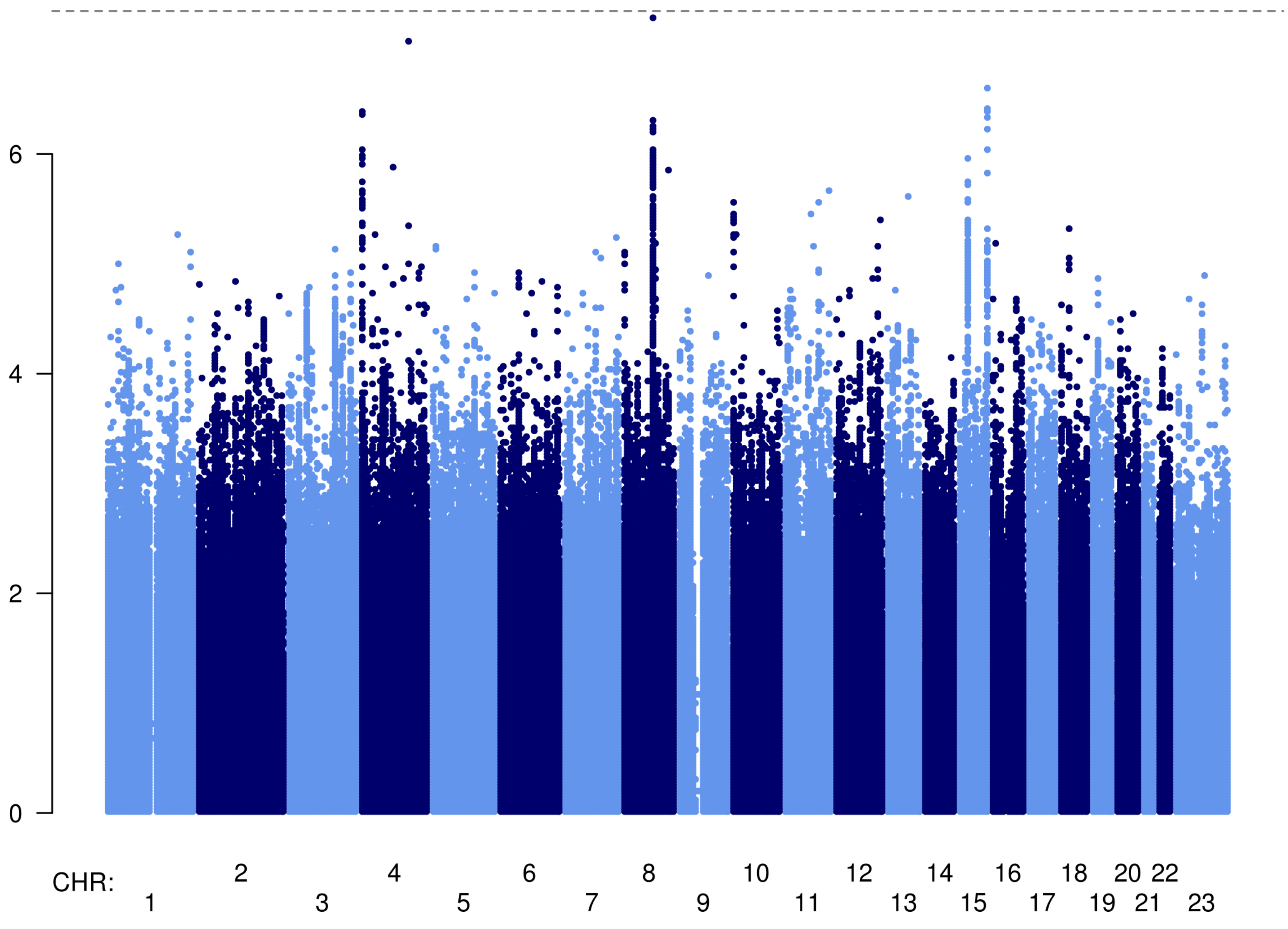

6
4
2
0
CHR:
1
2
3
4
5
6
7
8
9
10
11
12
13
14
15
16
17
18
19
20
21
22
23

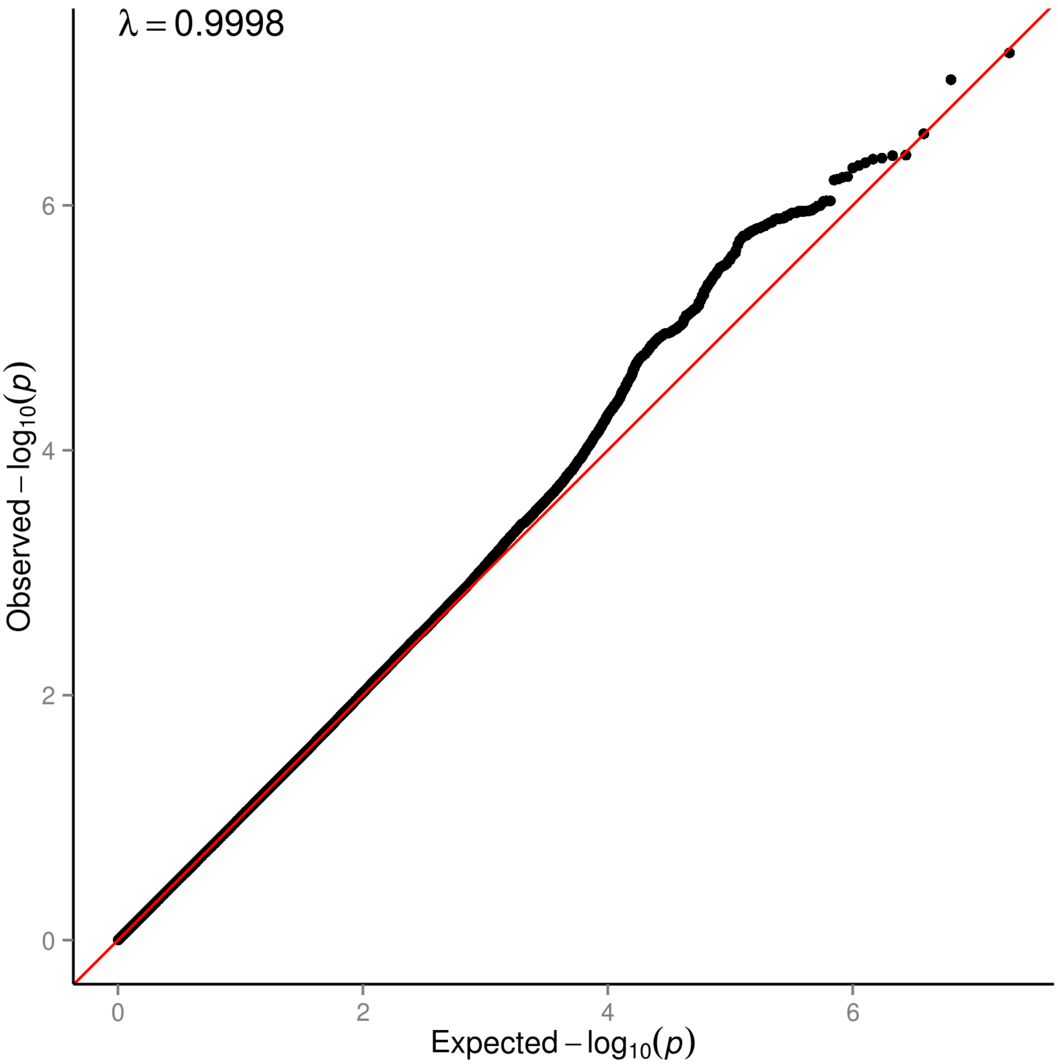

λ = 0.9998
Observed −log10(p)
Expected −log10(p)
0
2
4
6
0
2
4
6

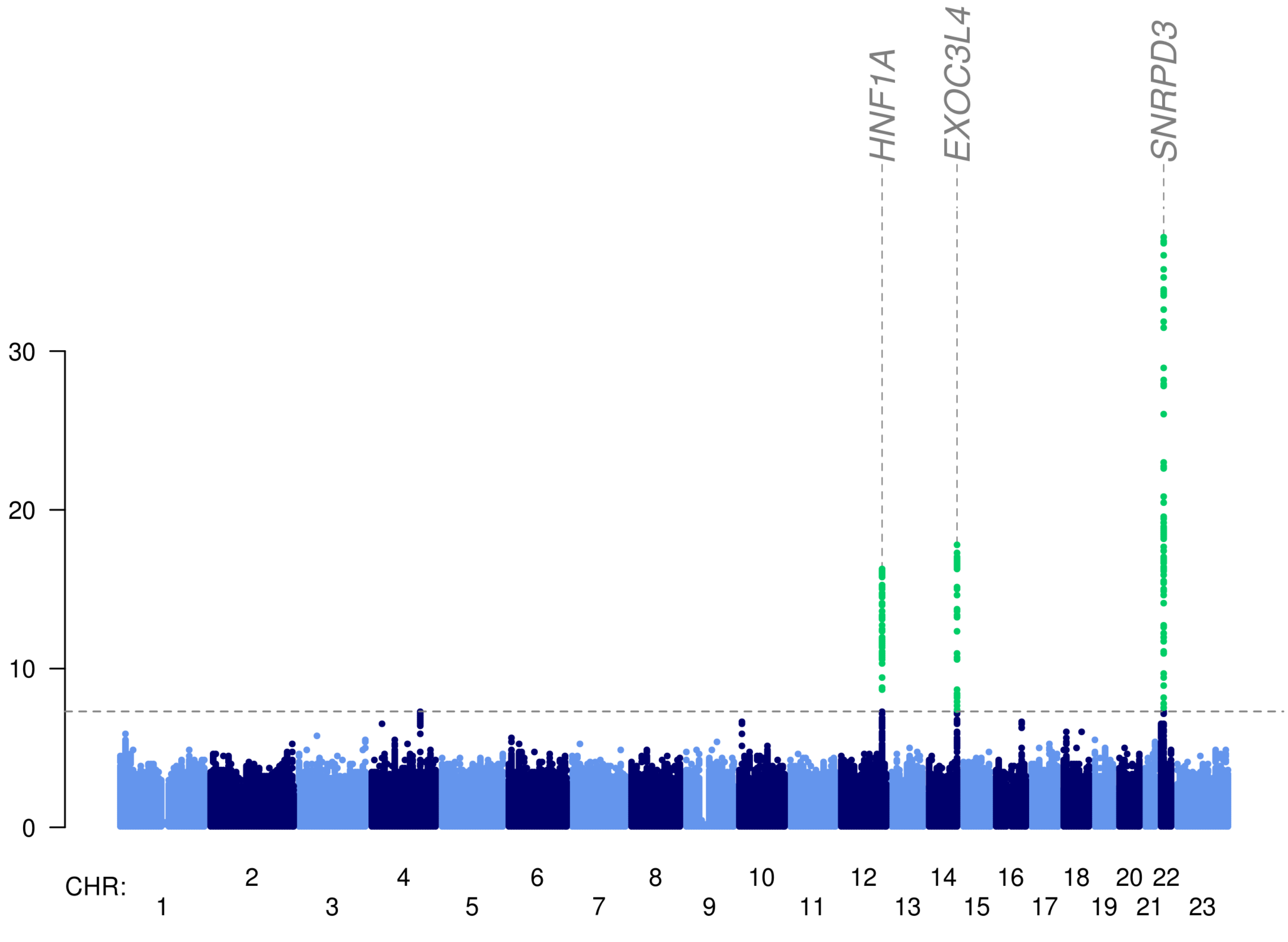
HNF1A
EXOC3L4
SNRPD3
30
20
10
0
CHR:
1
2
3
4
5
6
7
8
9
10
11
12
13
14
15
16
17
18
19
20
21
22
23

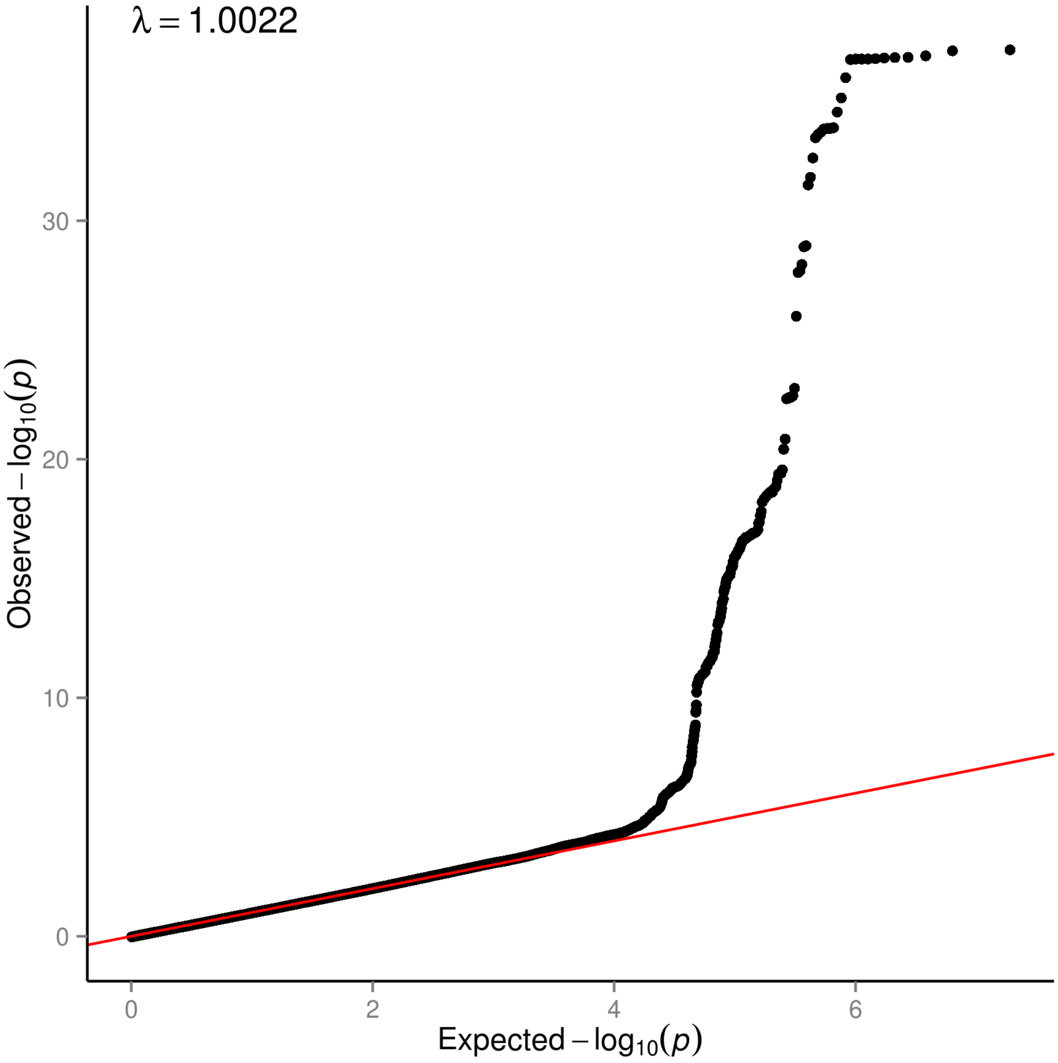

λ = 1.0022
Observed −log10(p)
Expected −log10(p)
0
10
20
30
0
2
4
6

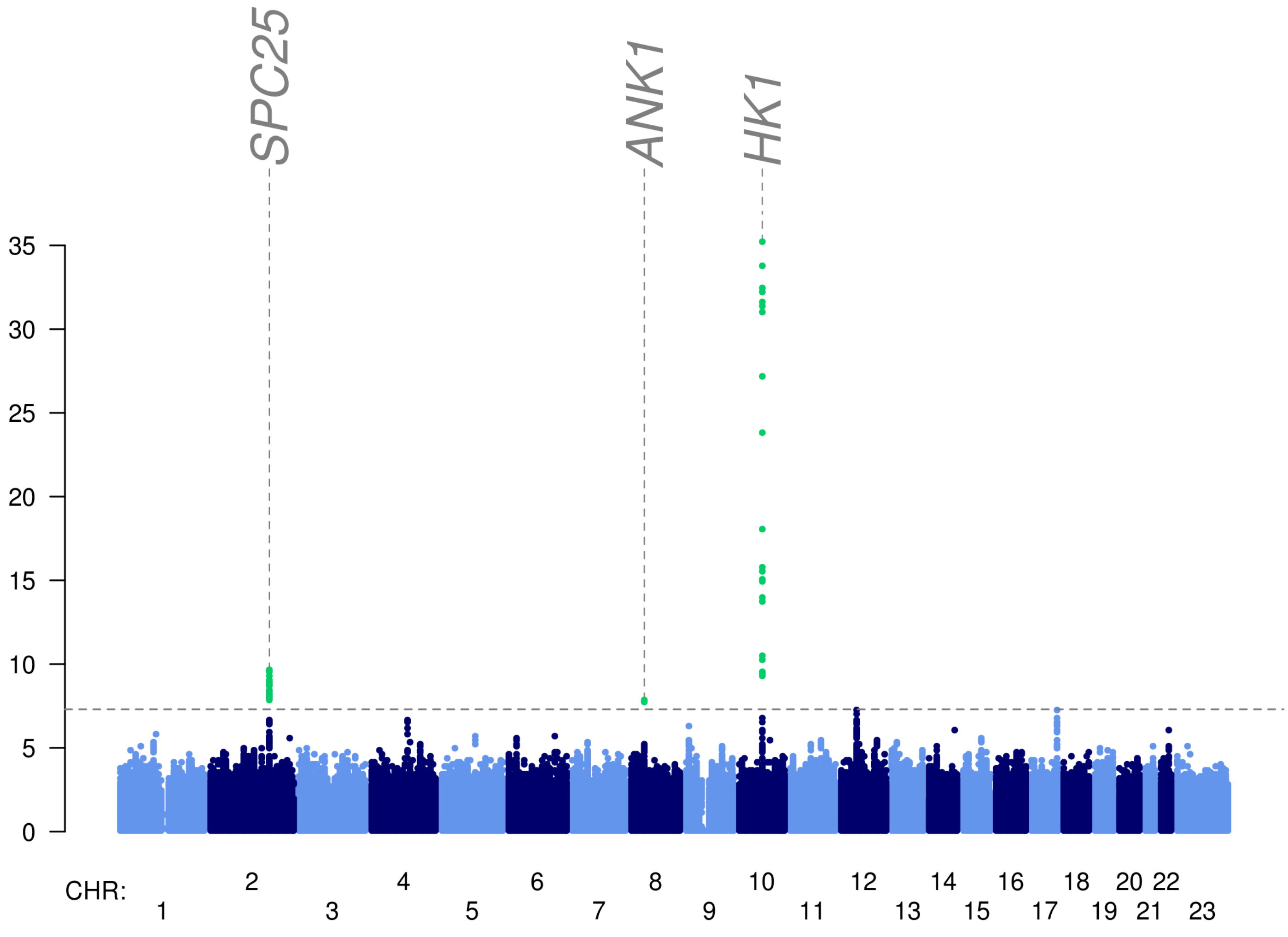
SPC25
ANK1
HK1
35
30
25
20
15
10
5
0
CHR:
1
2
3
4
5
6
7
8
9
10
11
12
13
14
15
16
17
18
19
20
21
22
23

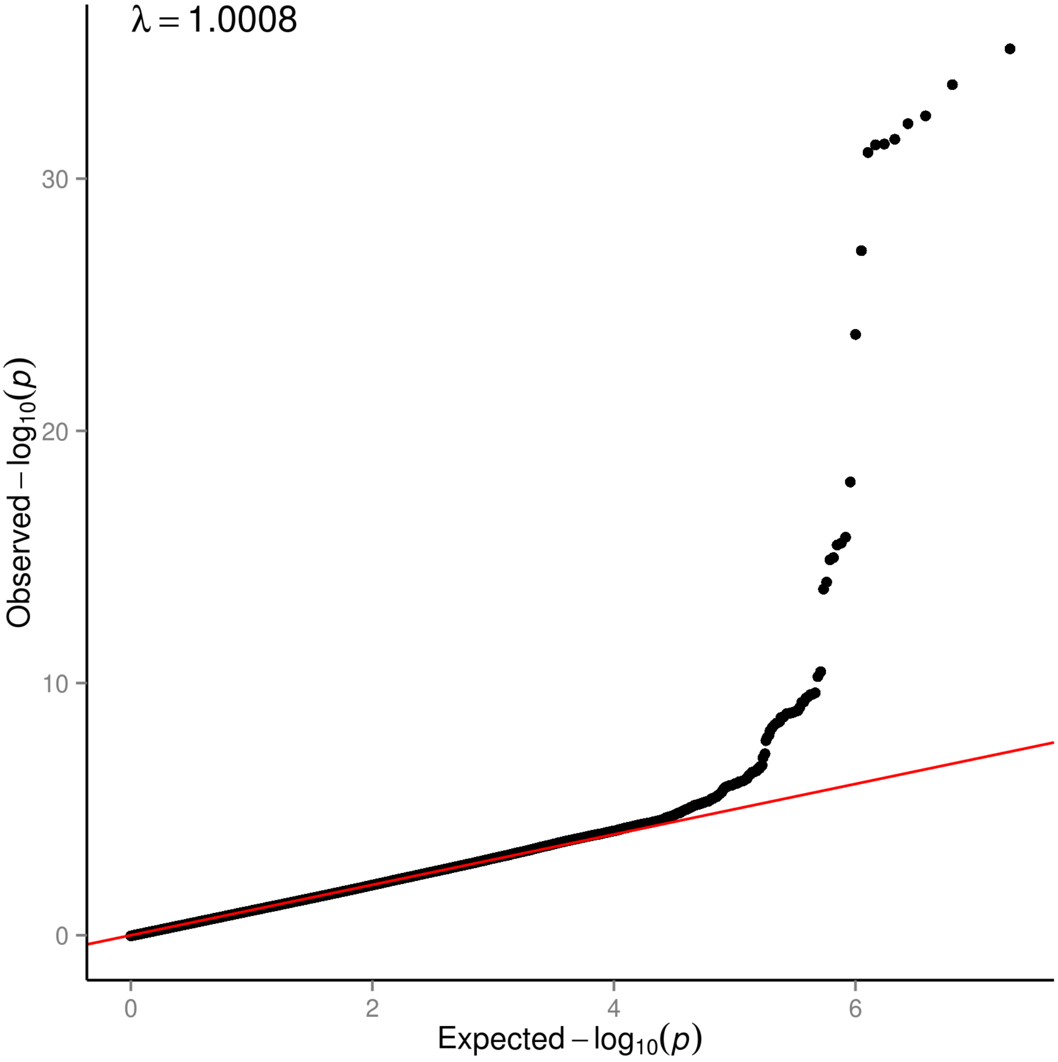

λ = 1.0008
Observed − log10(p)
Expected − log10(p)
0
10
20
30
0
2
4
6

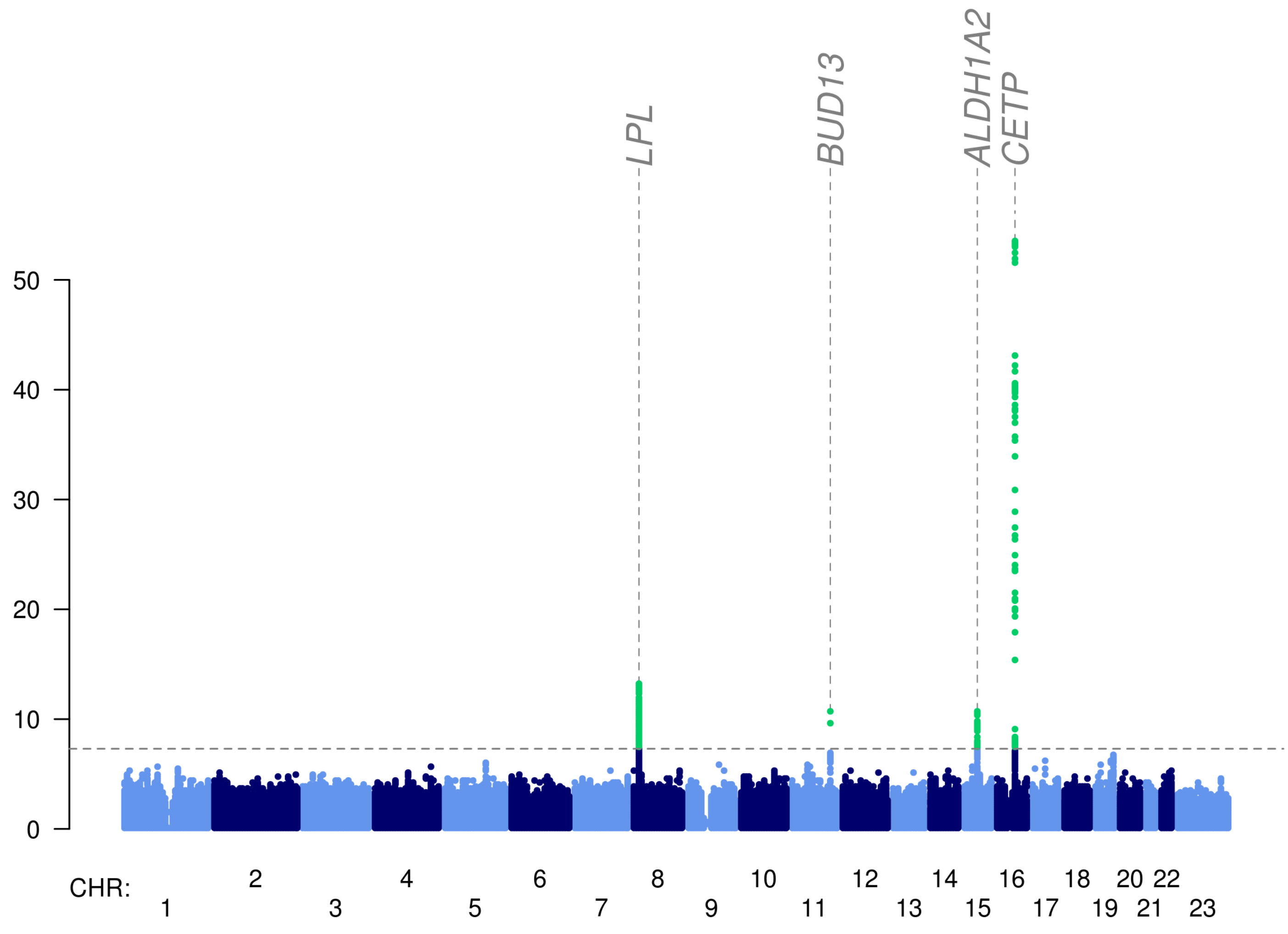
LPL
BUD13
ALDH1A2
CETP
50
40
30
20
10
0
CHR:
1 2 3 4 5 6 7 8 9 10 11 12 13 14 15 16 17 18 19 20 21 22 23

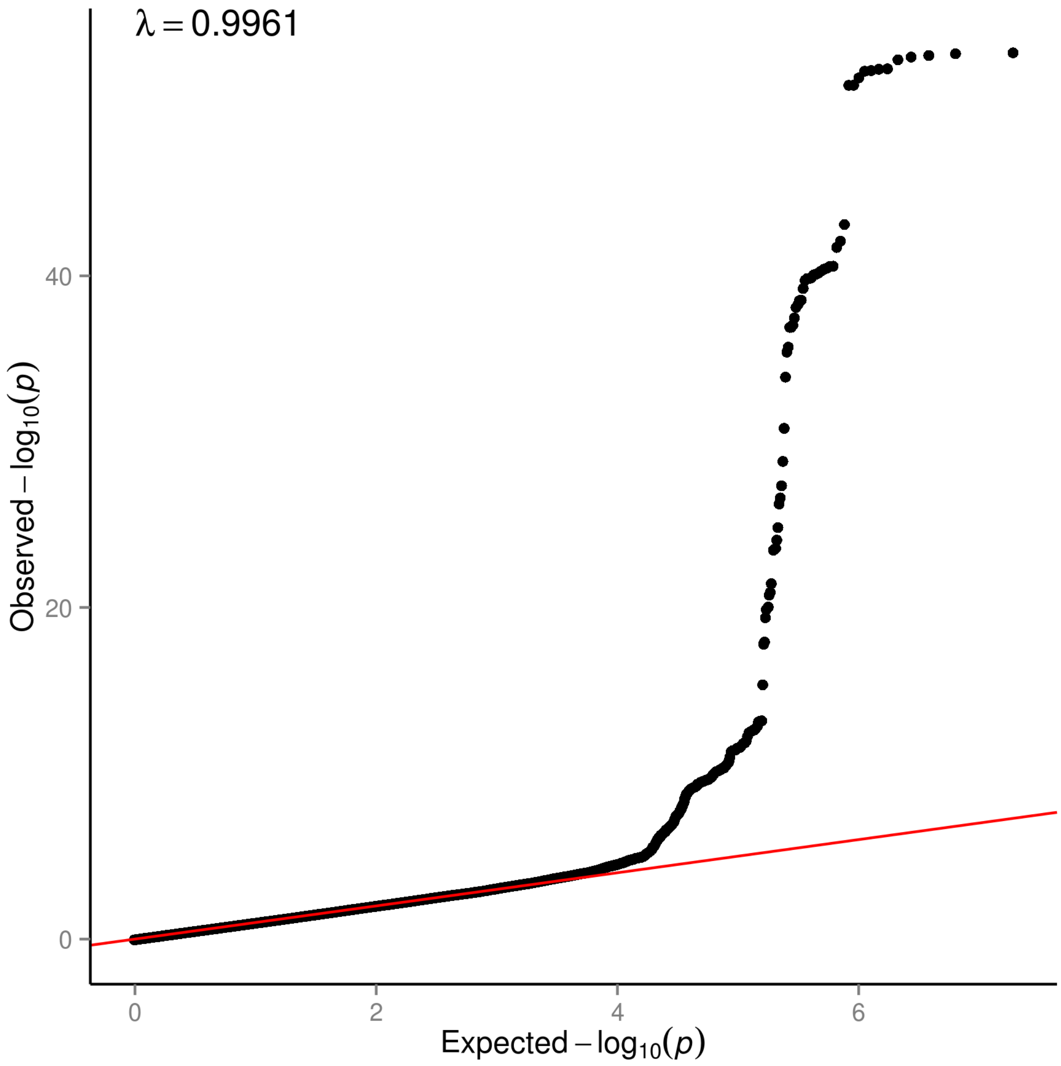

λ = 0.9961
40
20
0
Observed − log10(p)
0
2
4
6
Expected − log10(p)

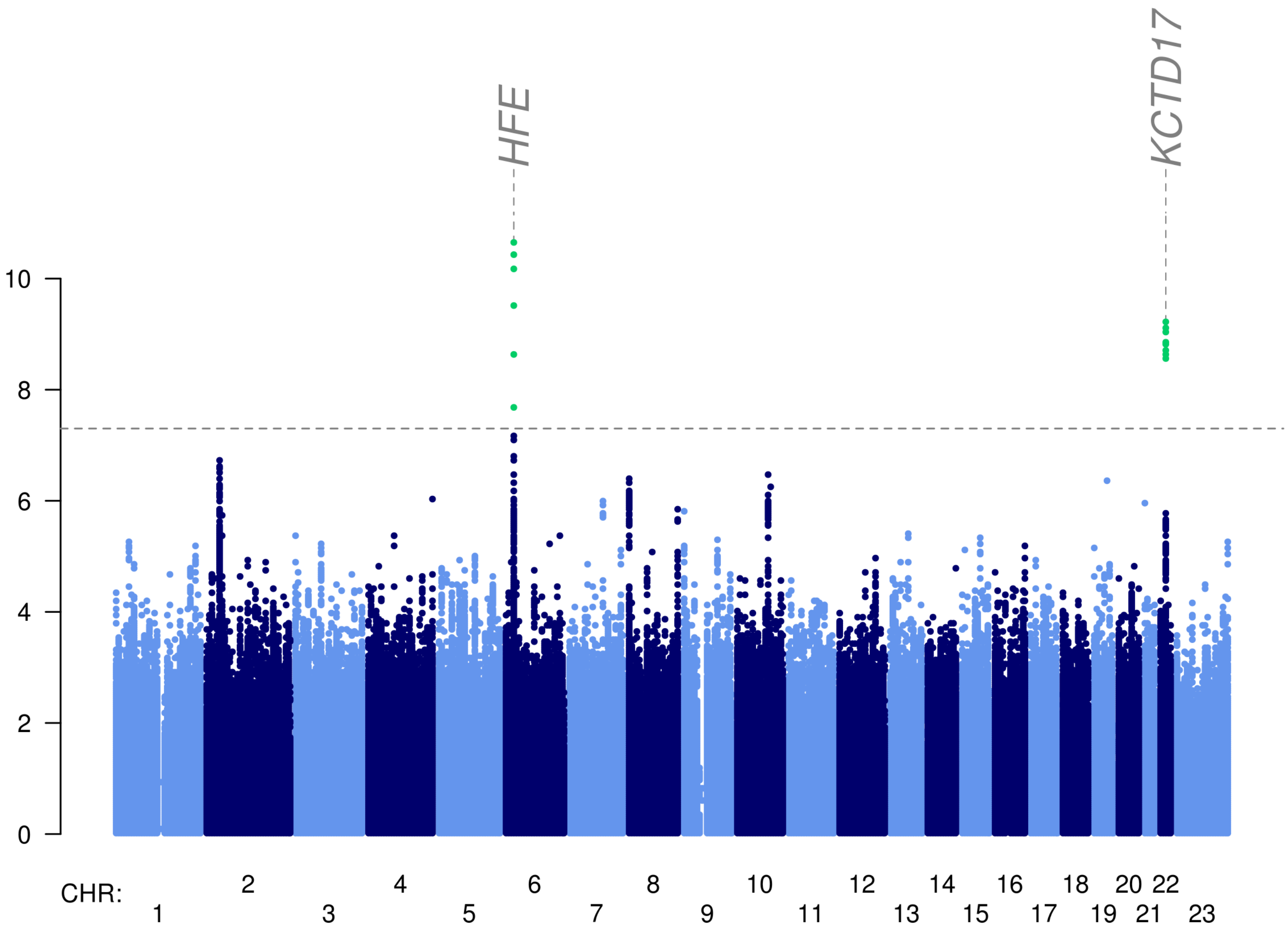
HFE
KCTD17
10
8
6
4
2
0
CHR:
1
2
3
4
5
6
7
8
9
10
11
12
13
14
15
16
17
18
19
20
21
22
23

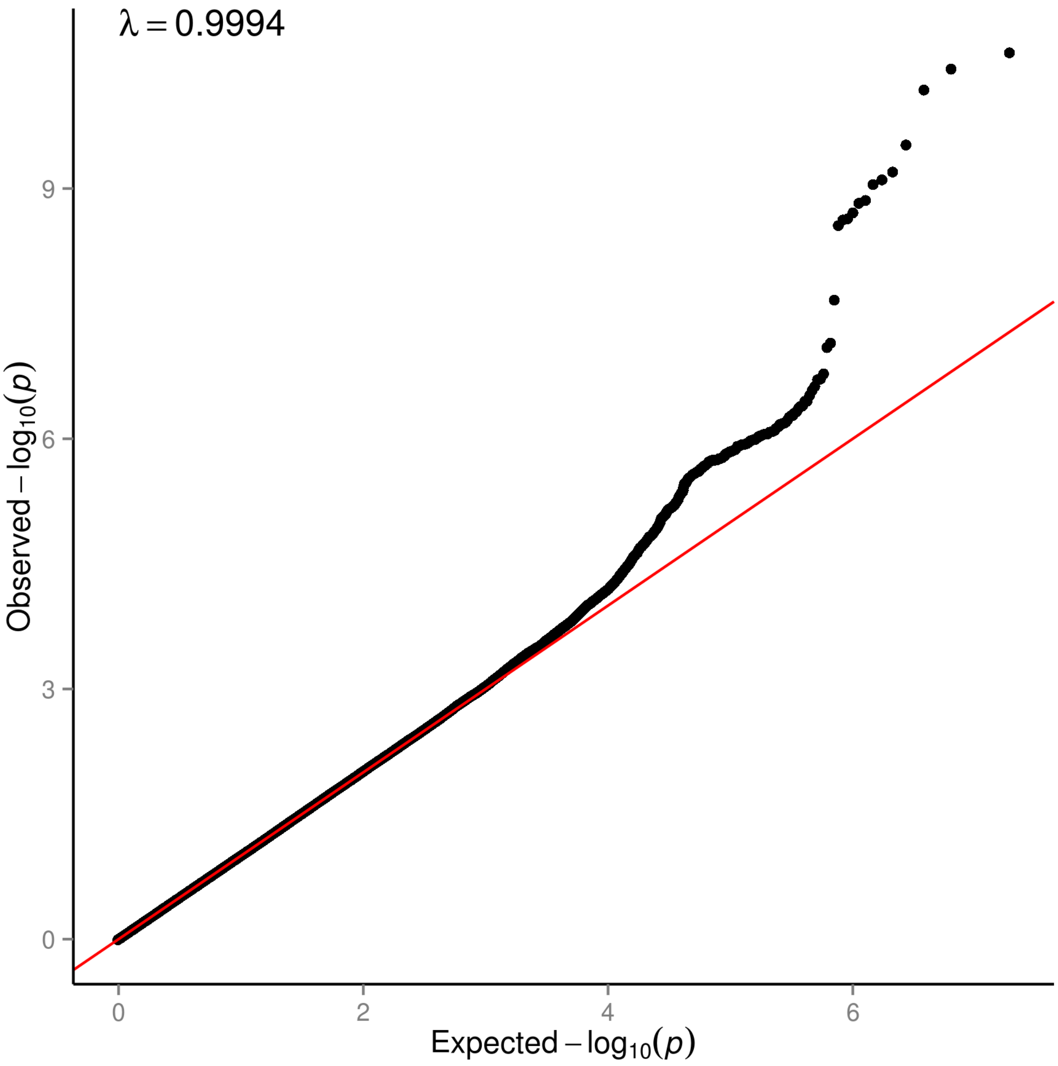

λ = 0.9994
Observed − log10(p)
Expected − log10(p)
0
3
6
9
0
2
4
6

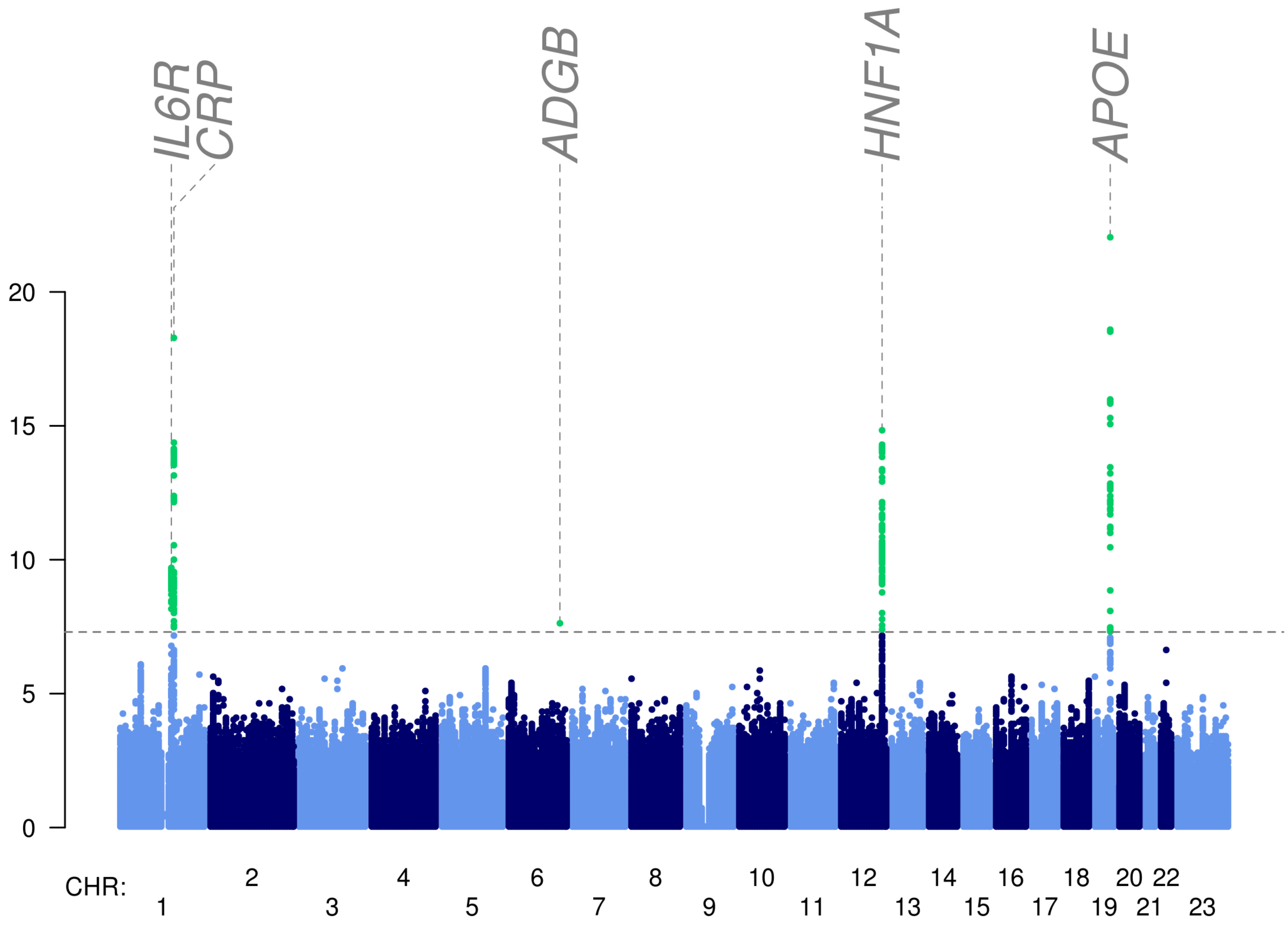
IL6R
CRP
ADGB
HNF1A
APOE
20
15
10
5
0
CHR:
1
2
3
4
5
6
7
8
9
10
11
12
13
14
15
16
17
18
19
20
21
22
23

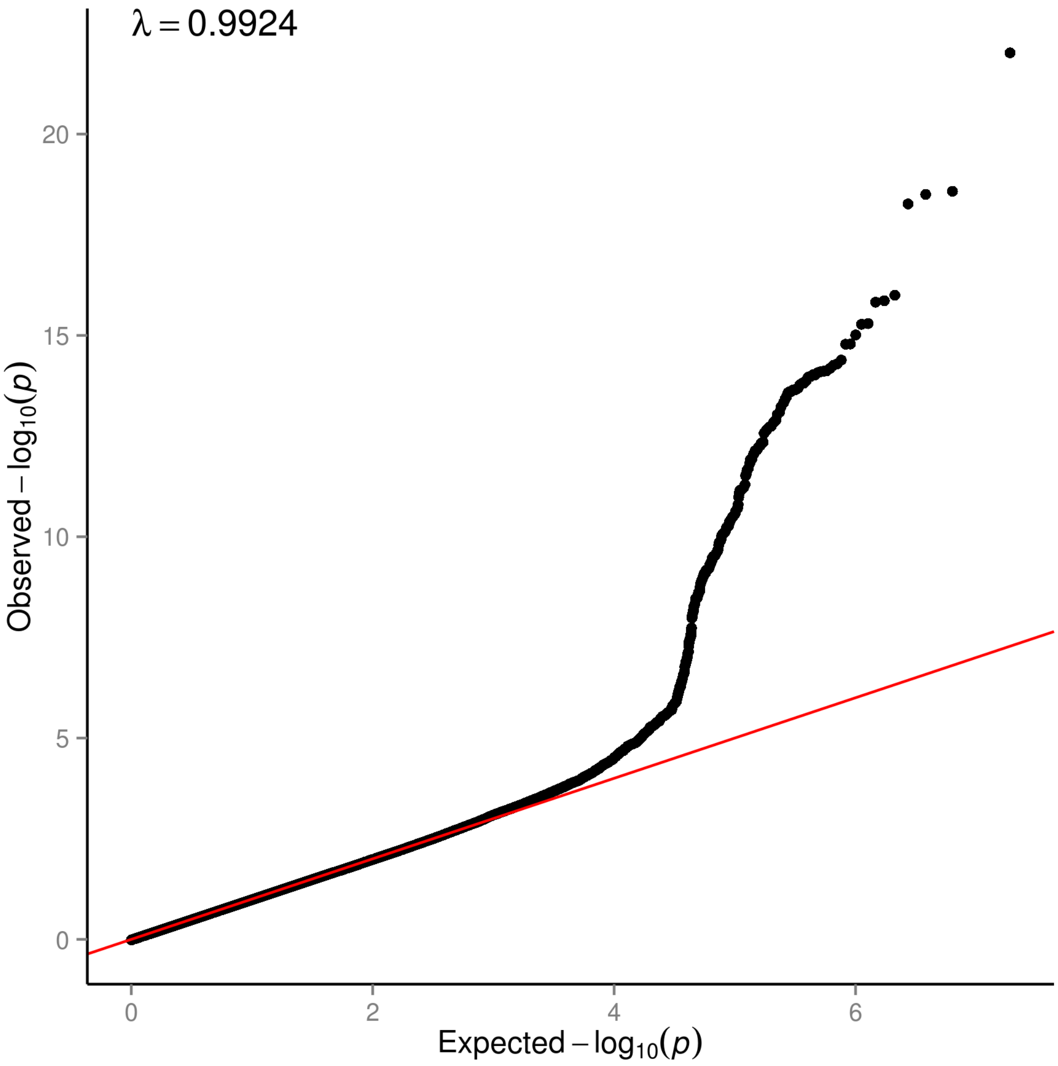

λ = 0.9924
Observed − log10(p)
Expected − log10(p)
0
5
10
15
20
0
2
4
6

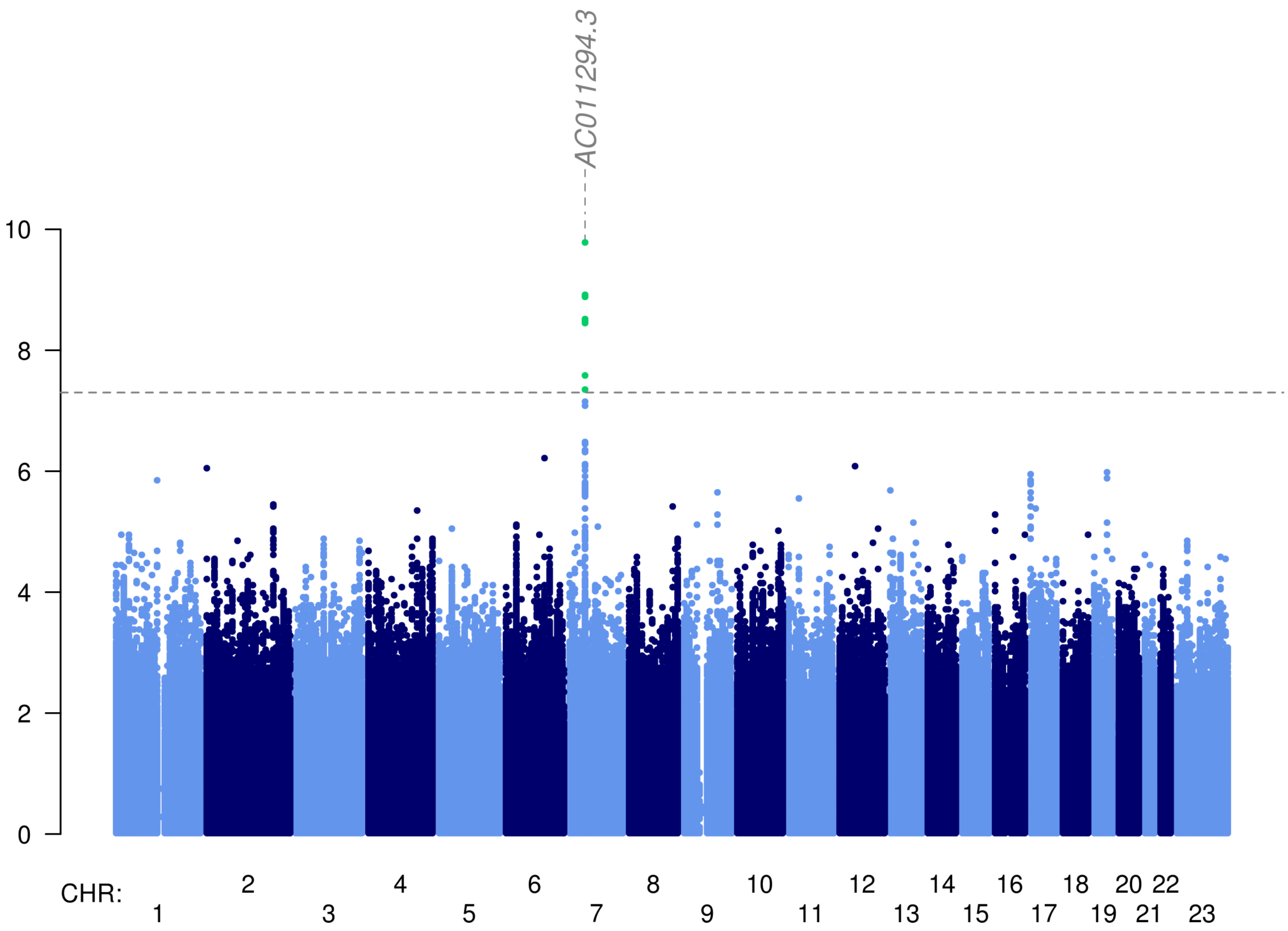
AC011294.3
10
8
6
4
2
0
CHR:
1
2
3
4
5
6
7
8
9
10
11
12
13
14
15
16
17
18
19
20
21
22
23

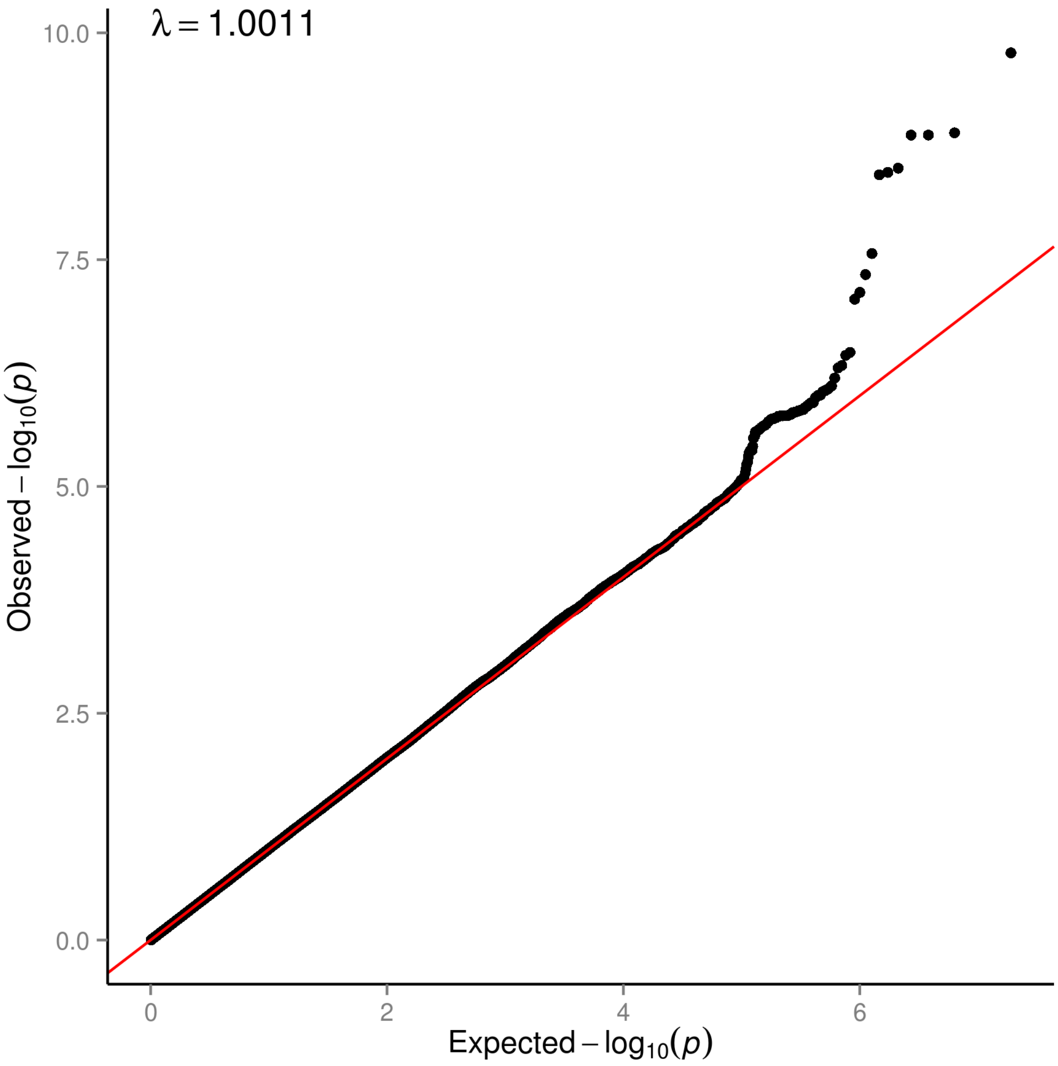
$\lambda = 1.0011$
10.0
7.5
5.0
2.5
0.0
0
2
4
6
Expected $-\log_{10}(p)$
Observed $-\log_{10}(p)$

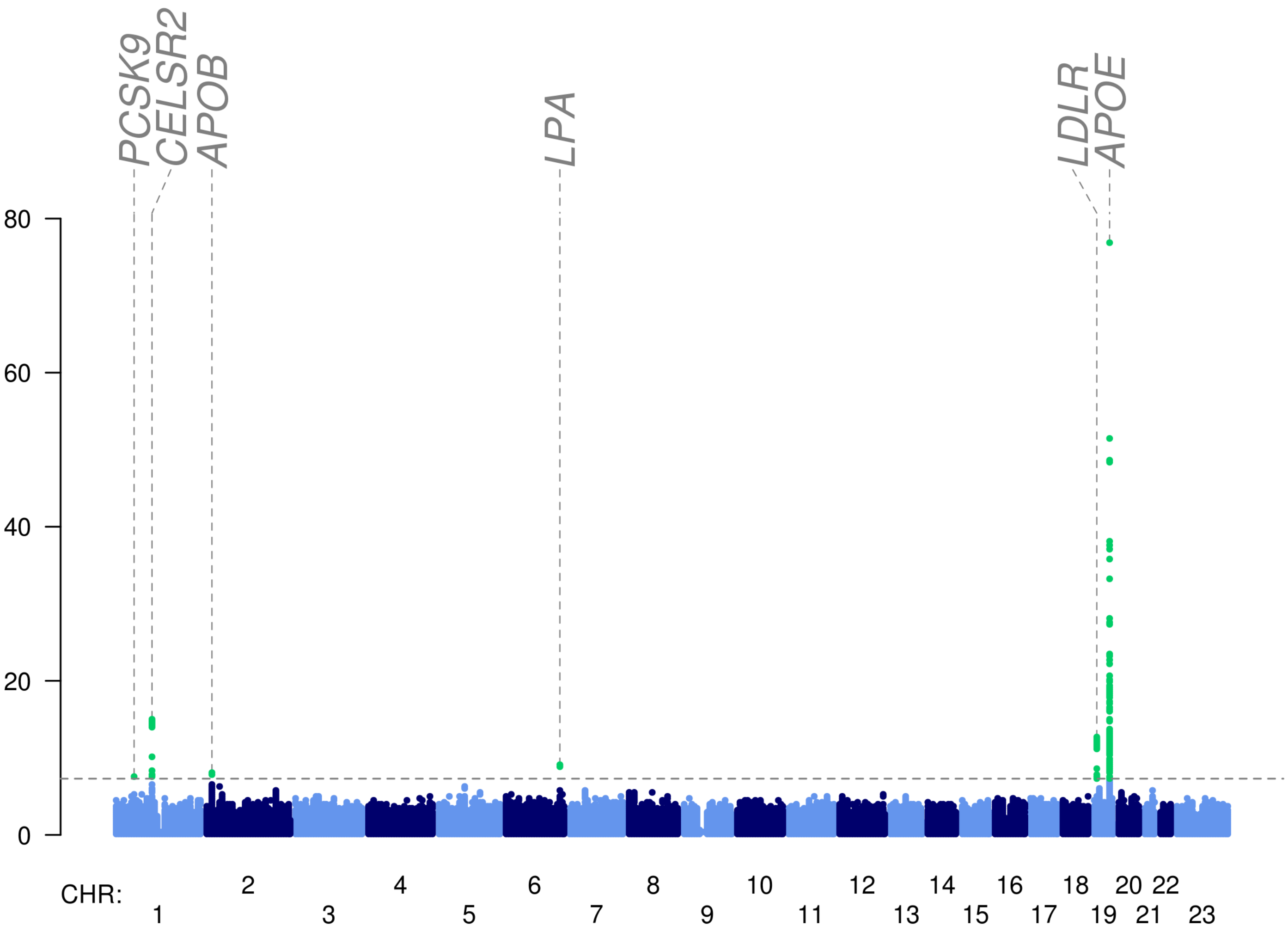
PCSK9
CELSR2
APOB
LPA
LDLR
APOE
80
60
40
20
0
CHR:
1 2 3 4 5 6 7 8 9 10 11 12 13 14 15 16 17 18 19 20 21 22 23

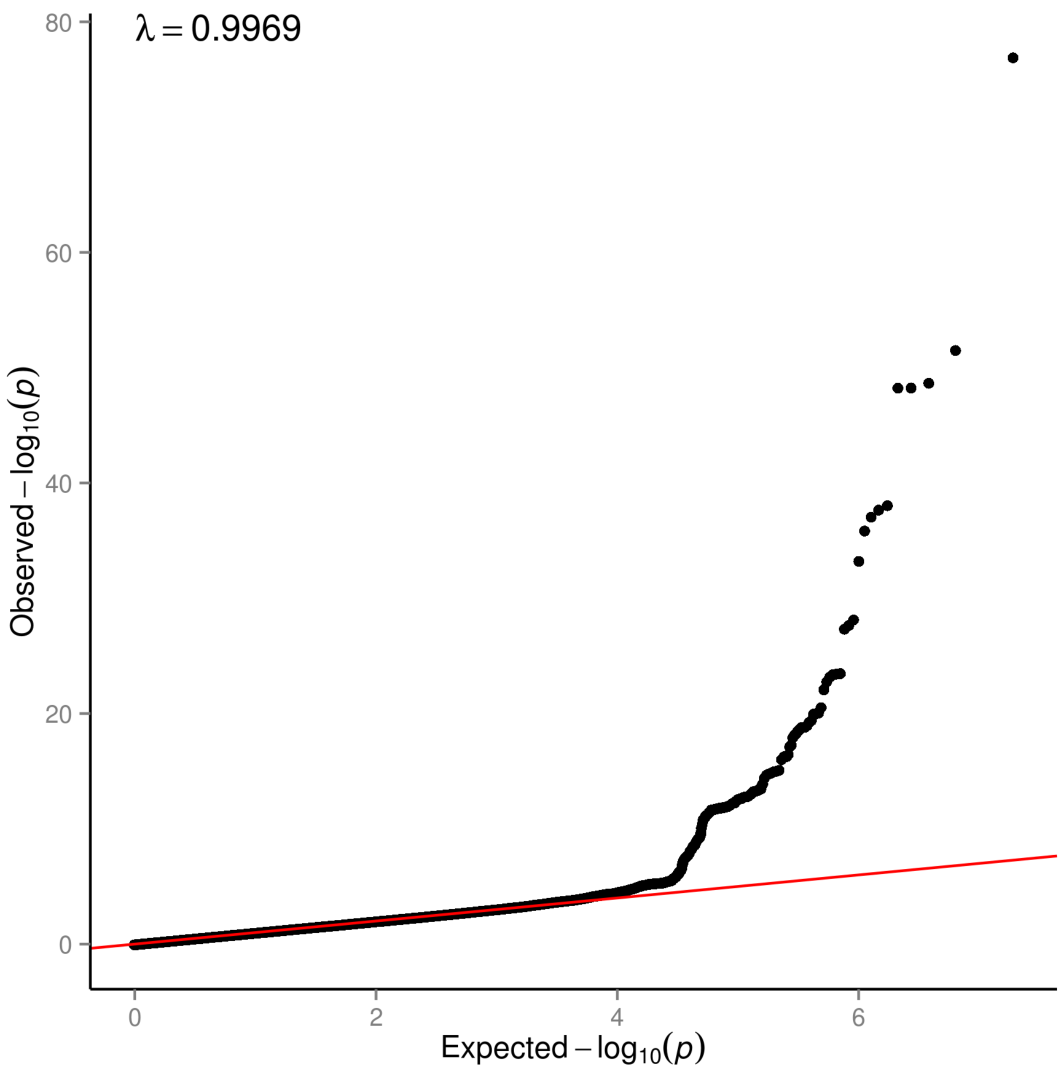

λ = 0.9969
Observed − log10(p)
Expected − log10(p)
0
20
40
60
80
0
2
4
6

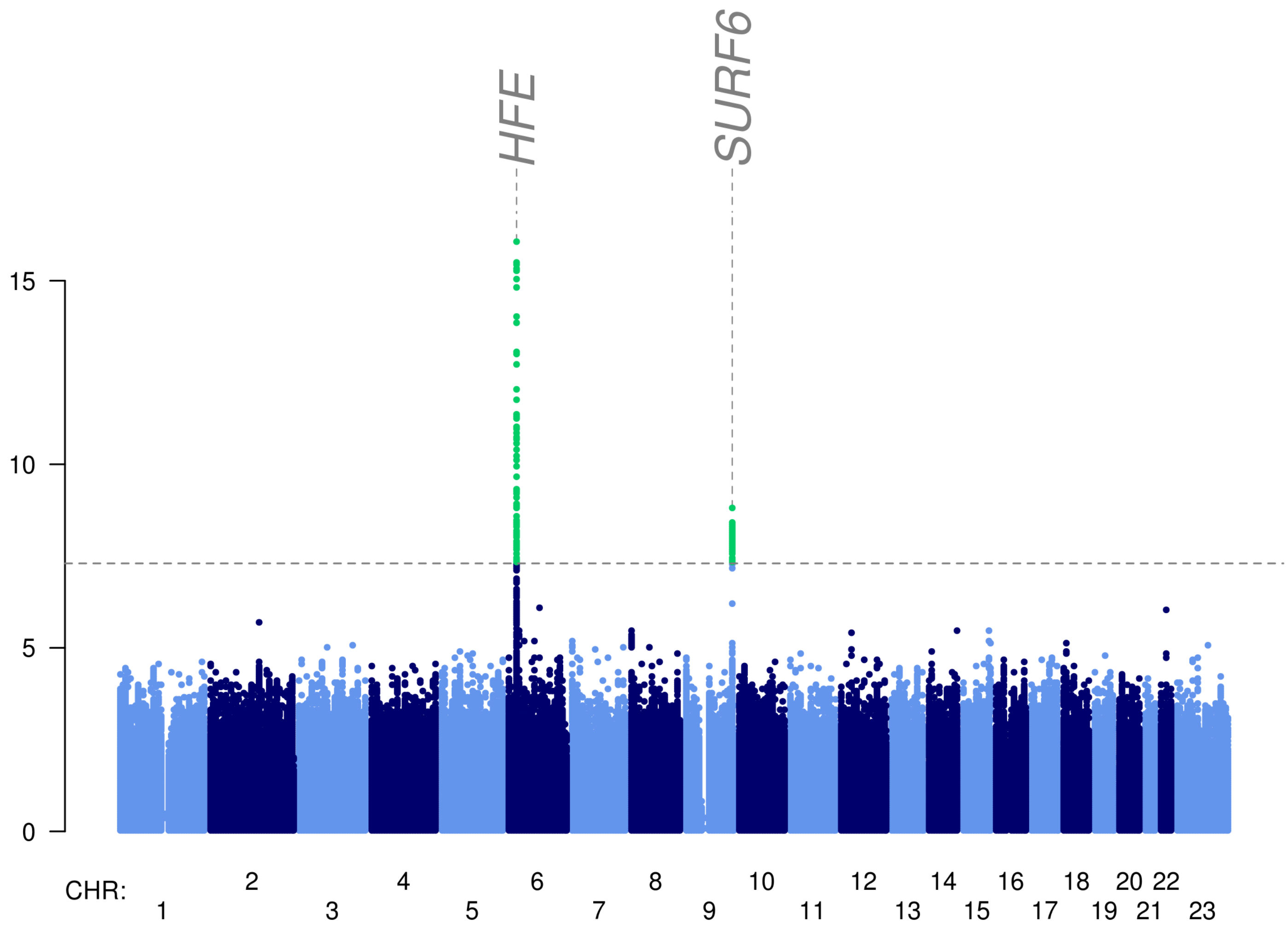
HFE
SURF6
15
10
5
0
CHR:
1
2
3
4
5
6
7
8
9
10
11
12
13
14
15
16
17
18
19
20
21
22
23

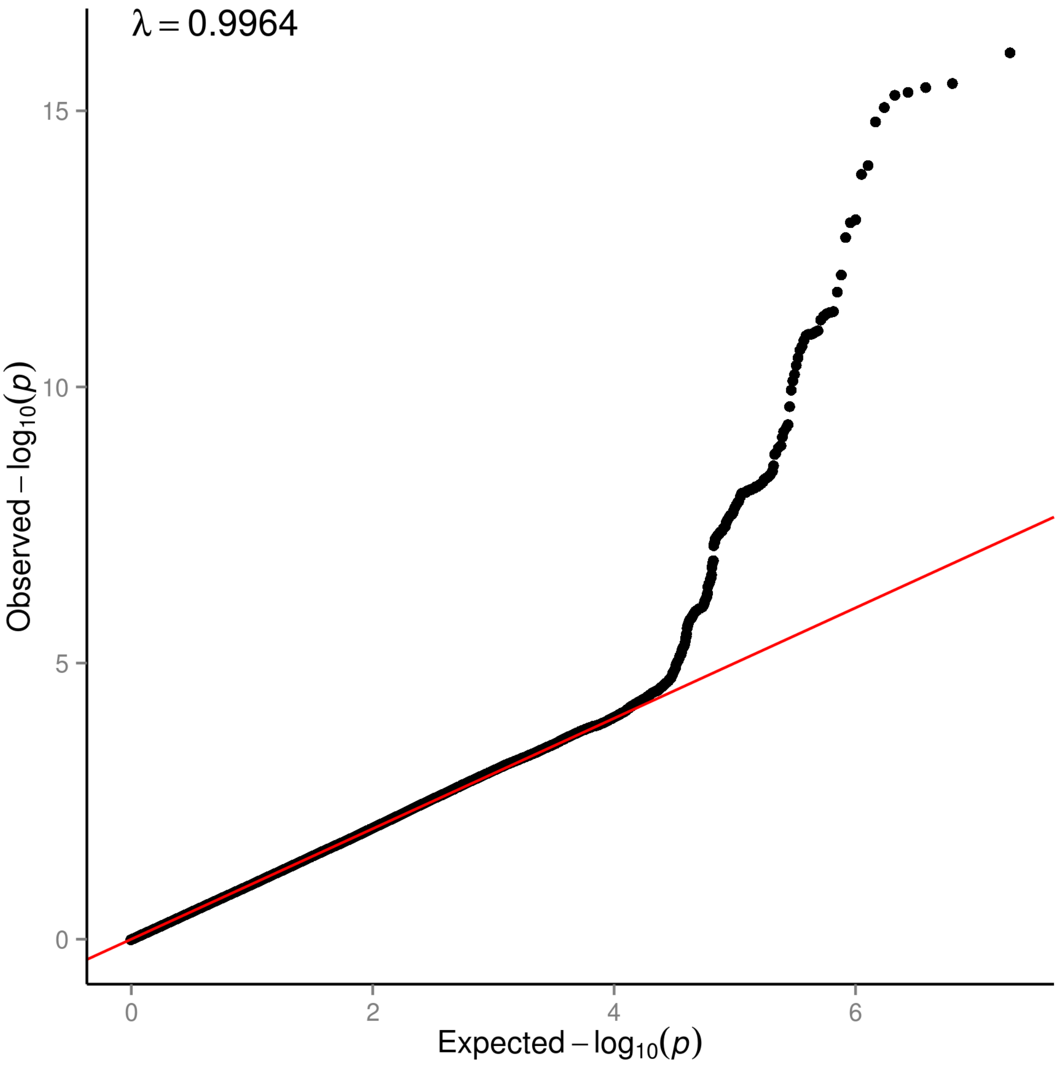
λ = 0.9964
15
10
5
0
Observed − log10(p)
0
2
4
6
Expected − log10(p)

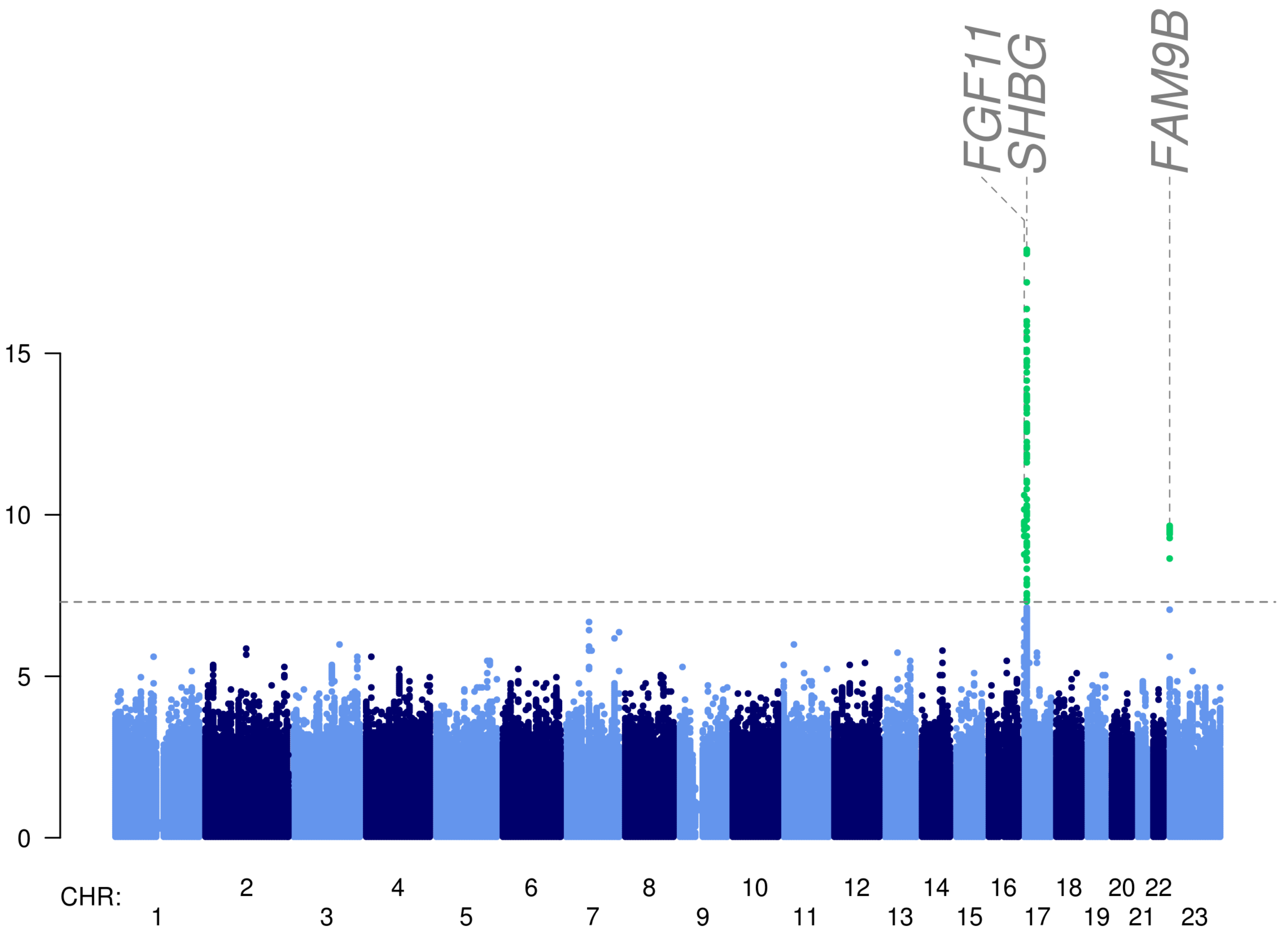
FGF11
SHBG
FAM9B
15
10
5
0
CHR:
1
2
3
4
5
6
7
8
9
10
11
12
13
14
15
16
17
18
19
20
21
22
23

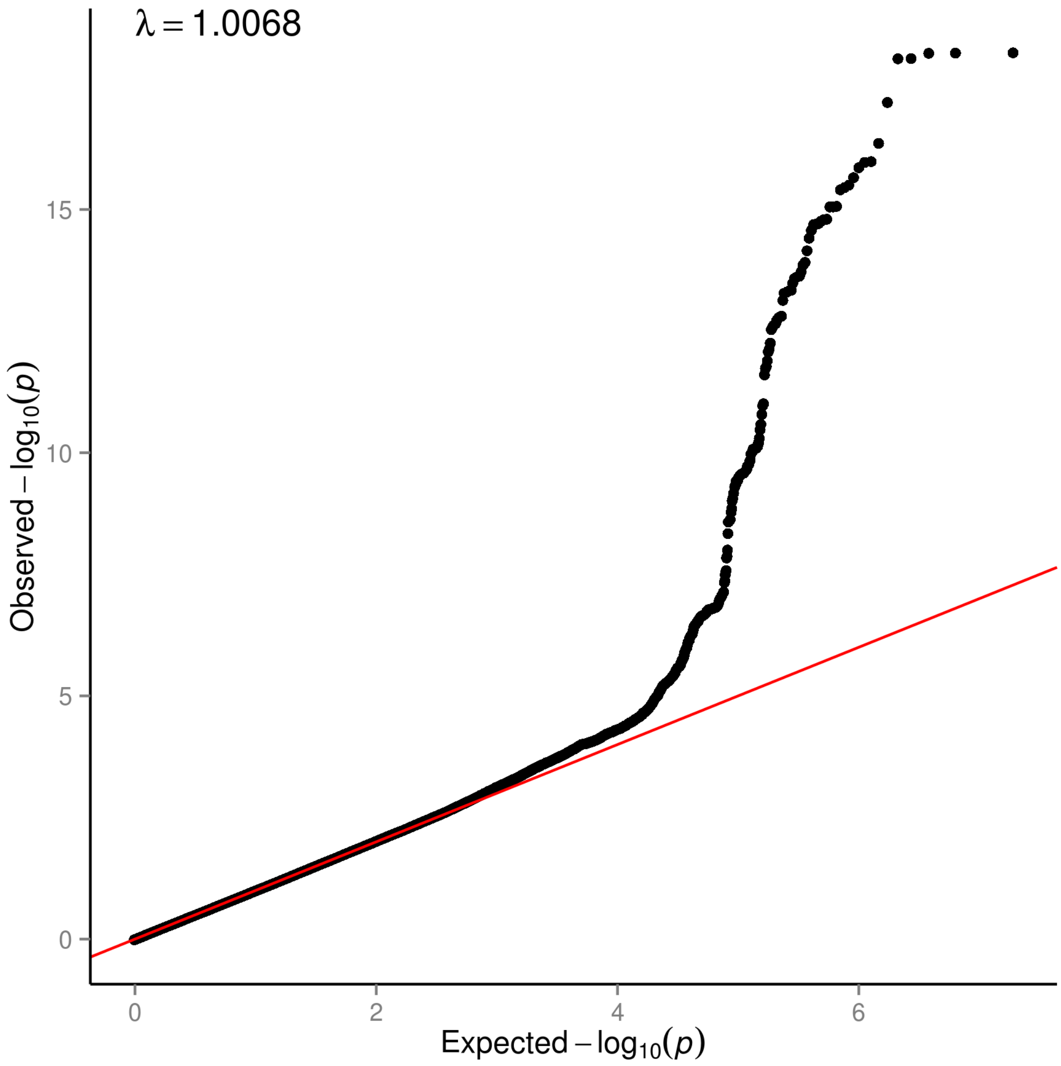

λ = 1.0068
Observed − log10(p)
Expected − log10(p)
0
5
10
15
0
2
4
6

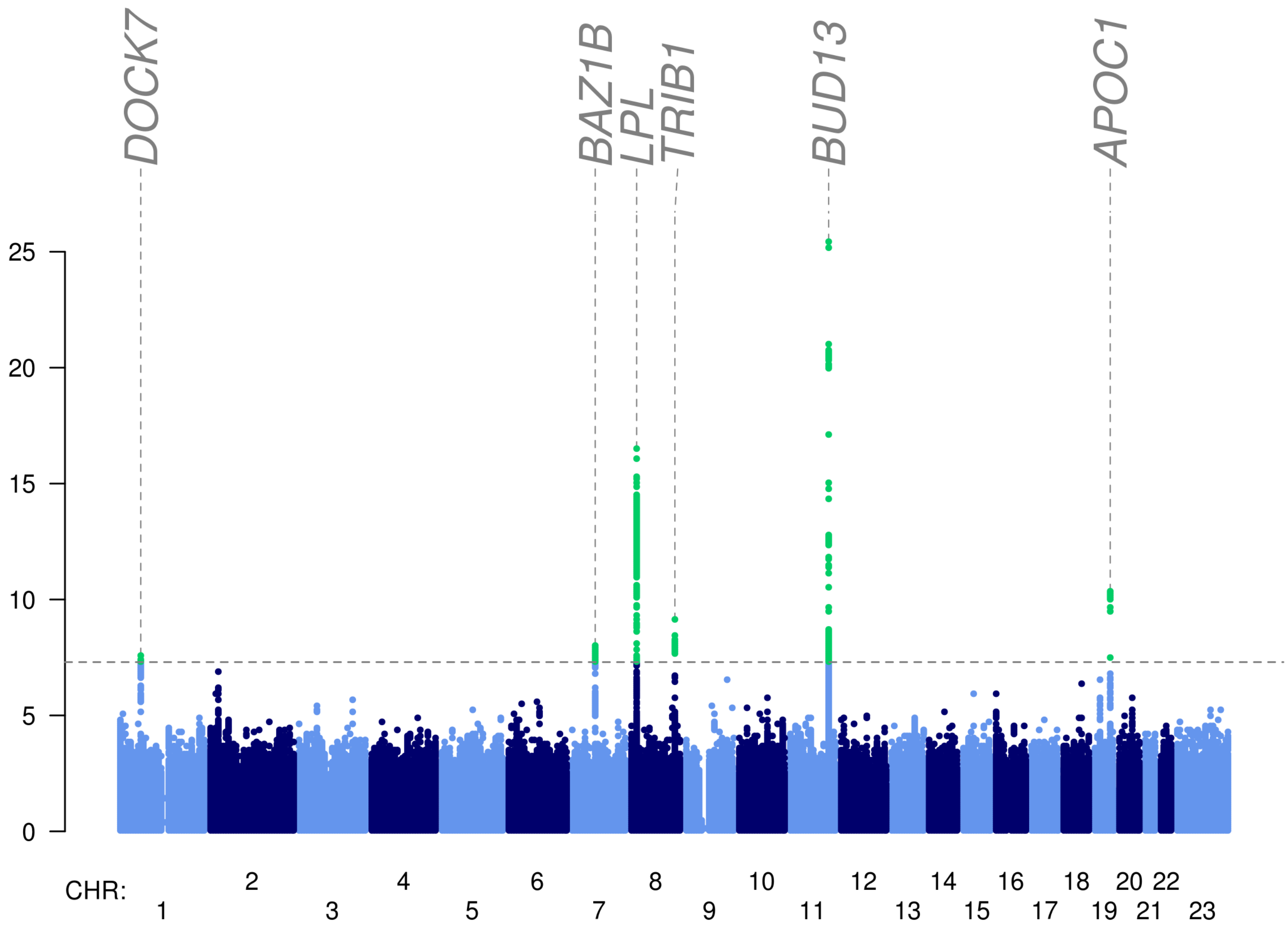
DOCK7
BAZ1B
LPL
TRIB1
BUD13
APOC1
25
20
15
10
5
0
CHR:
1
2
3
4
5
6
7
8
9
10
11
12
13
14
15
16
17
18
19
20
21
22
23

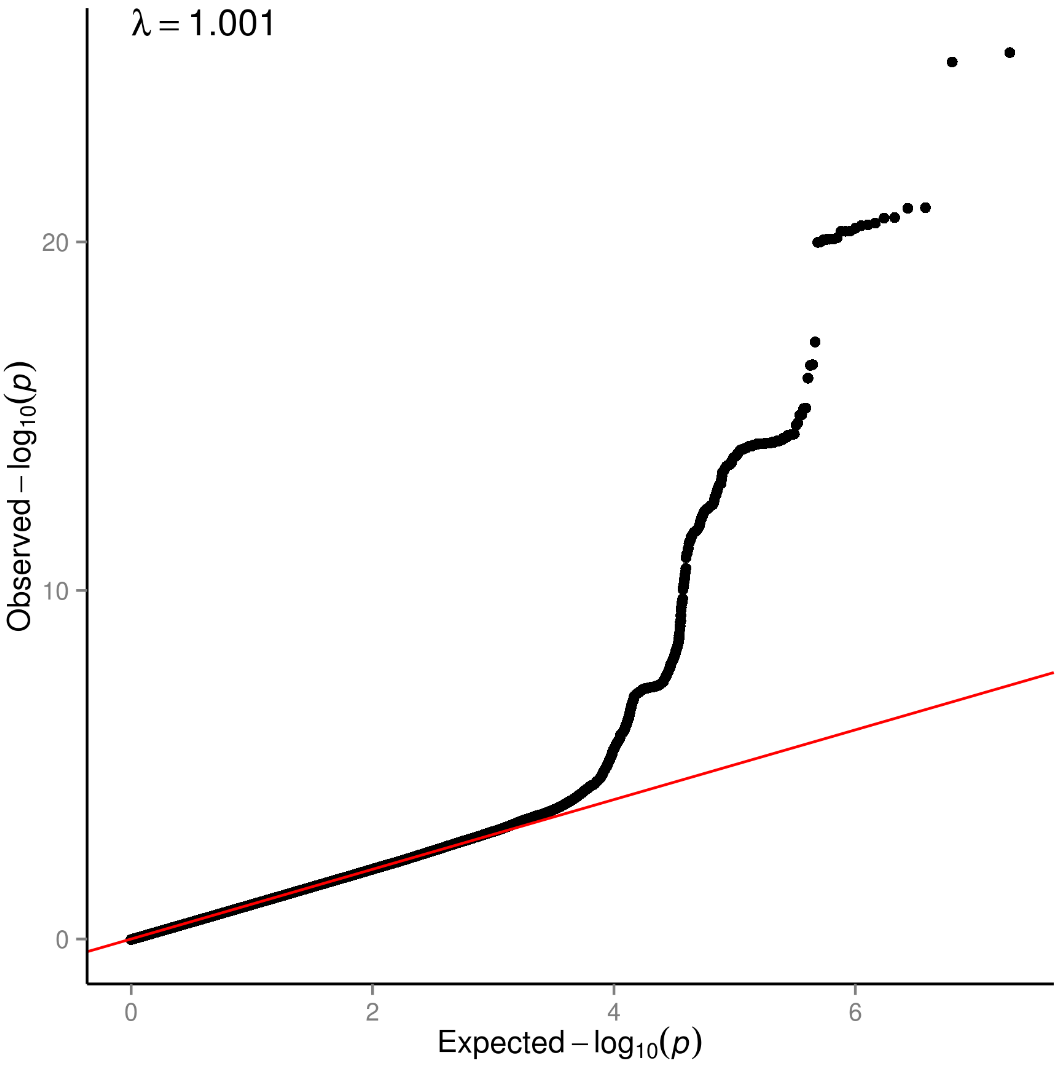
λ = 1.001
Observed − log10(p)
Expected − log10(p)
0
10
20
0
2
4
6

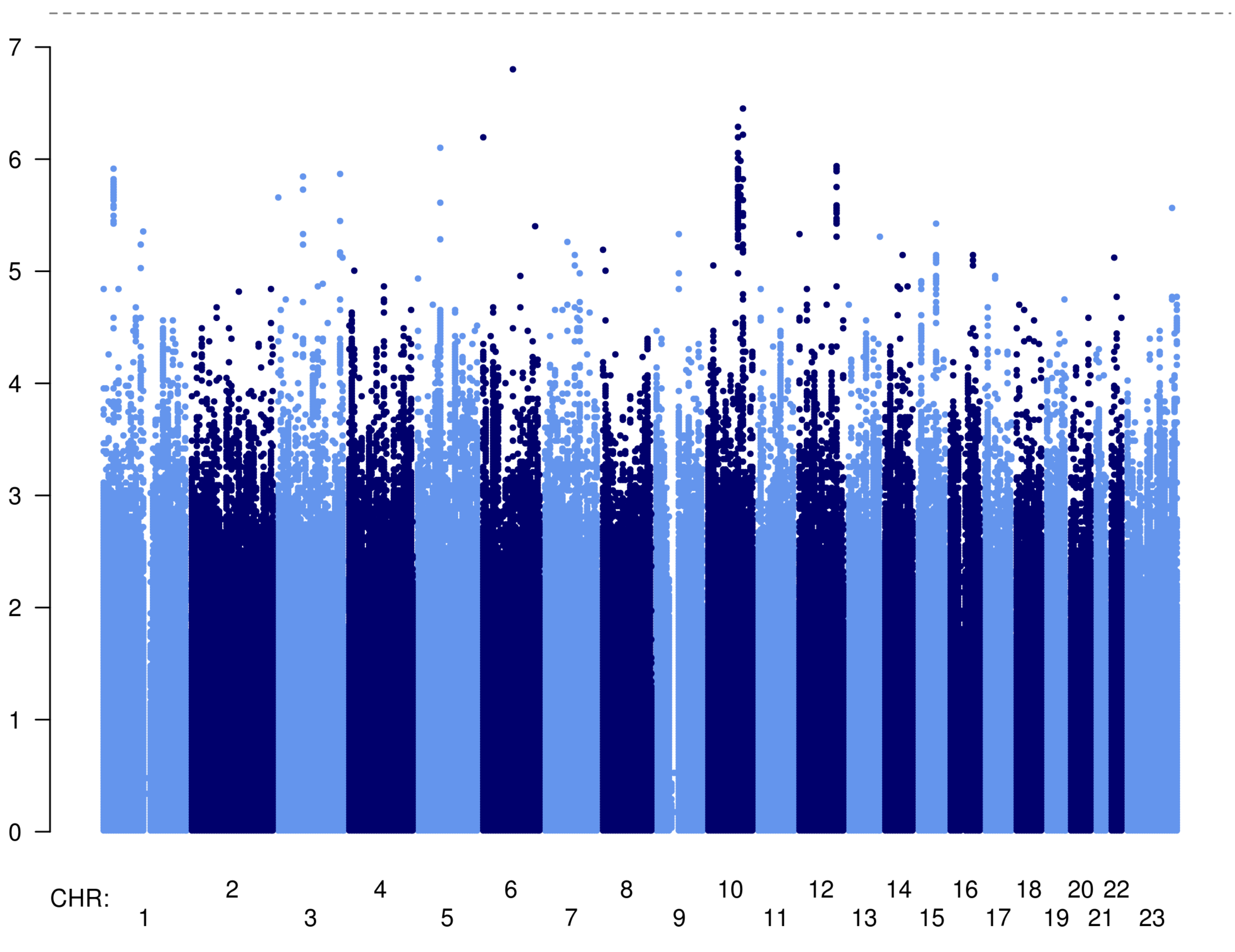

7
6
5
4
3
2
1
0
CHR:
1 2 3 4 5 6 7 8 9 10 11 12 13 14 15 16 17 18 19 20 21 22 23

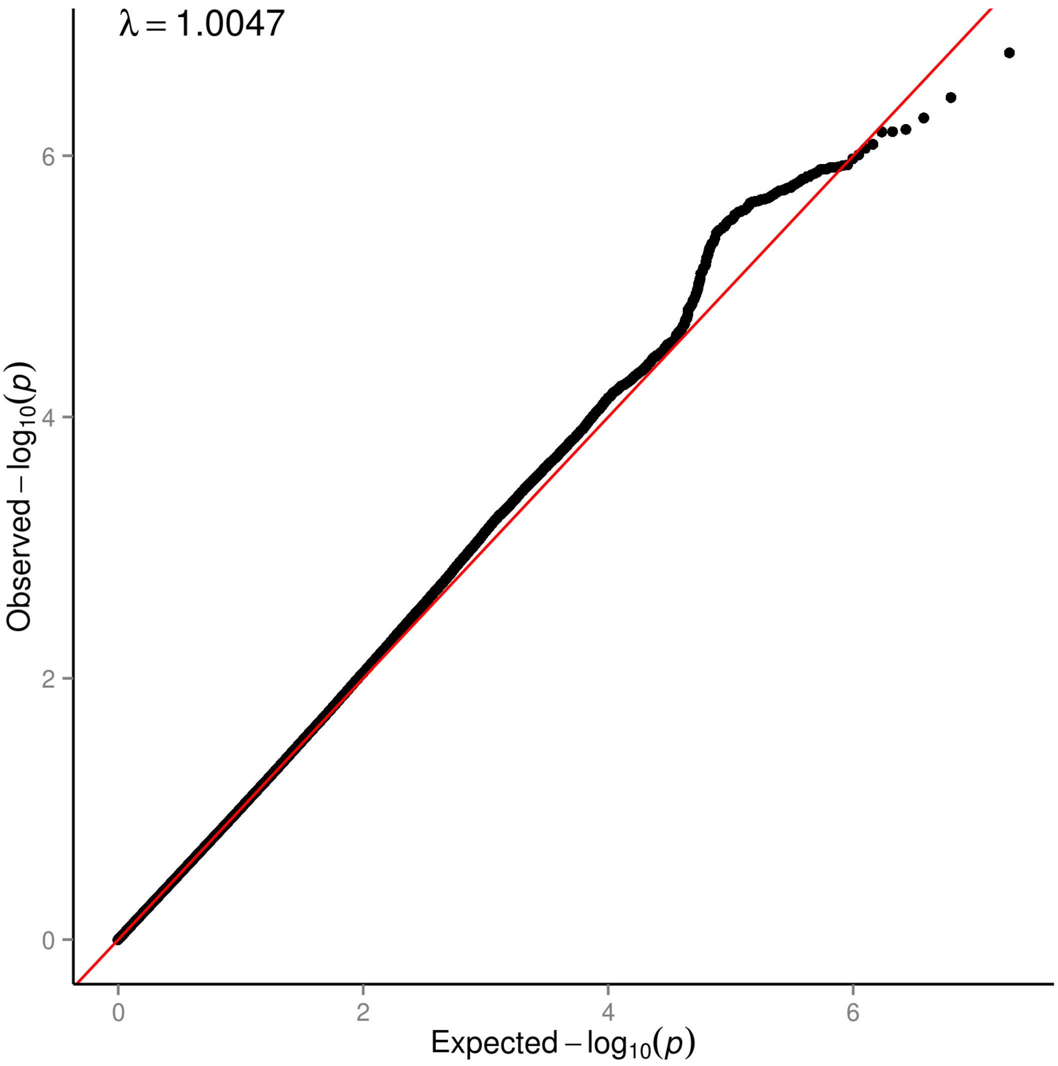

$\lambda = 1.0047$
Observed $-\log_{10}(p)$
Expected $-\log_{10}(p)$
0
2
4
6
0
2
4
6
